# The effect of axial geometrical variations on the steady state characteristics of oil lubricated journal bearings using titanium dioxide nanoparticles as lubricant additives

**DOI:** 10.1038/s41598-025-97948-7

**Published:** 2025-05-05

**Authors:** H. Awad, Khaled M. Abdou, E. Saber

**Affiliations:** Arab Academy for Science, Technology and Maritime Transport, Alexandria, Egypt

**Keywords:** Journal bearings, Nanolubricant, Nanoparticle, Aggregate particle size, Engineering, Nanoscience and technology

## Abstract

The current work examines journal bearings with axial geometrical configurations that are lubricated with nanofluids. Because of recent advancements in numerically controlled machine tools, accurate machining of complex shapes is now a realistic operation. A theoretical prediction of bearing characteristics for different complicated geometries with varying bearing length to shaft diameter ratios at varying eccentricity ratios is required prior to any experimental effort. The Reynolds like equation that governs the pressure distribution inside the bearing is provided. Four various bearing geometries with conical (wedge), concave, convex, and wavy surfaces are chosen to investigate the bearing characteristics while taking into account the impact of increasing nanoparticle concentrations and aggregate particle sizes. The modified Krieger–Dougherty viscosity model was used to investigate the influence of TiO_2_ nanoparticle lubricant additives on the performance of the chosen journal bearings. The results show that the chosen shapes improve the bearing’s performance over the plain cylindrical bearing. The concave design is also shown to be better than the others; however the findings show that an optimization method may be required to acquire the geometry that provides the optimum bearing characteristics.

## Introduction

Hydrodynamic journal bearings are in high demand nowadays because of their excellent characteristics such as long-term performance, low friction, and nearly no wear. There are two types of research articles: numerical studies and experimental studies. Numerical investigations are further characterized as isothermal studies with cavitation, isothermal studies without cavitation, and investigations that include thermal effects. More than 75% of the study is done theoretically utilizing numerical/computational approaches, while approximately 23% of the experiments have been carried out. In the literature, attempts to study the effect of geometrical variations on the performance of journal bearings have been reported in different manners. Some studies looked at non-circular geometrical shapes of the bearing bush, such as elliptical and multiple lobes^1–8^. Others investigated the influence of the unavoidable geometrical changes generated by either angular misalignment or journal deflection^9–13^. Leung et al.^14^ investigated journal bearings using spherical bushes. They concluded that the spherical journal bearing behaves similarly to a comparable cylindrical bearing. El-Gamal^15^ on the other hand, published a study for a cylindrical wedge-shaped bearing. His findings revealed that the wedge bearing at small wedge angles, particularly the long one, is better than the plain cylindrical bearing with the same side length. Pang et al.^16^ used AG, a genetic algorithm, to optimize the shape of hydrodynamic journal bearings in circumferential and axial wavy configurations. The Fourier series coefficients and axial waviness serve as design parameters in both circumferential and axial directions. Their findings indicated that the load capacity may be increased by around 10%. Green tribology aims to reduce friction and wear. Surface texturing on mating parts can minimize friction while increasing load capacity. Surface textures with various microgeometries, including dimples, grooves, and pits, have been proven to increase bearing performance. Textured bearing surfaces minimize friction while increasing load capacity. Researchers^17–22^ evaluated the influence of textural parameters such as the shape, depth, and dimensions of microgeometries, as well as their positions and distribution on the bearing surface, on journal bearing performance. Research results indicated that roughness characteristics and operating circumstances significantly impact journal bearing performance^20–22^. Currently, as computer technology advances, more researchers are attempting to use commercial computational fluid dynamics (CFD) applications in their researches. The CFD code differs from other comparable codes in that it uses all of the Navier–Stokes equations to solve complex flow problems, whereas finite difference systems depend on the Reynolds equation. Furthermore, the CFD packages are applicable in highly complicated geometries. Many authors employed various computer codes to examine the parametric influence of hydrodynamic journal bearing, and their results are listed in Table 1.

**Table 1:** Summary of articles employing commercial computer codes.

Ref	Technique	Bearing type	Parameter	Analysis/Results
^23^	CFD	Journal bearing with smooth and textured surface	Surface texture, eccentricity ratio and friction force	Condition of light loading reduced frictional force and increased minimum film thickness; under heavy loading circumstances, a larger pressure zone reduces frictional force
^24^	CFD	Central circumferential groove of hydrodynamic journal bearing	Bearing carrying capacity, cavitation zone and vapor fraction	The depth of the groove influences the load zone, bearing carrying capacity, cavitation zone, and vapor fraction
^25^	CFD and FSI	Hydrodynamic journal bearing	length to diameter ratio, eccentricity ratios, and pressure	The FSI technique is utilized to find the pressure, stress, and deformation of the hydrodynamic journal bearing
^26^	CFD	Journal bearing with bingham fluid	Eccentricity ratio, yield stress	Fluent software examined real and theoretical data for Newtonian and Bingham lubricants and found good agreement. The influence of yield stress on the journal bearing is similarly evaluated to be minor for low eccentricity ratios
^27^	CSD and CFD	Full $360^{o}$ journal bearing	Deformation and stress distribution	According to the article, these strategies are helpful for The finite element method (FEM) was utilized to calculate the stress distribution. Determine the surface deformation of the bearing under static load. The effects of resulting forces are also examined. The modeling of elasto-hydrodynamic lubrication has been validated with standard lubrication results
^28^	CFD-FSI	Journal bearing	Deformation, eccentricity ratios and speeds	Develop models for various eccentricity ratios and speeds to investigate the relationship between the elastic behavior of the bearing and the fluid. This procedure produced accurate performance of the bearing
^29^	CFD and FSI	Thermo-hydrodynamic and thermo-elastohydrodynamic analysis of full journal bearing	Pressure, temperature and velocity distribution in the fluid film, and bearing surface deformation	The characteristics under static load conditions are determined using the finite volume and finite element methods. The distortion caused by pressure is an essential variable in determining bearing behavior
^30^	COMSOL models	Hydrodynamic bearing	Pressure distribution, eccentricity ratio	Pressure distribution is determined on infinite (short and long) bearings under steady state conditions. It was expected that growing pressure is proportional to eccentricity ratio, and pressure increases in the direction of eccentricity
^31^	ANSYS, MATLAB software	Bush type journal bearing	Temperature	It is shown that there are approximately 12% differences between the two procedures. However, Ansys provided a more exact result than the numerical method.
^32^	CFD	Journal bearing	Pressure, temperature viscosity, L/D ratio, rotational speed, Eccentricity ratio, pressure distribution	Software results were validated using numerical data obtained from the Raimondi and Boyd chart approach. It has been proposed that increasing temperature raises pressure while reducing attitude angle
^33^	CFD (Gambit and using fluent 6.3.26 )	Plain journal bearing	Pressure distribution, temperature and viscosity	It is noticed that increasing frictional force increases the temperature, lowers viscosity, and the maximum pressure of the lubricant
^34^	CFD	Circular journal bearing	Pressure and temperature distribution	When the viscosity is held constant, temperature and pressure increase
^35^	CFD and FSI	Infinitely long journal bearing	Pressure and temperature variation	It was determined that maximum pressure occurred closer to the region of a minimum film thickness
^36^	CFD	Journal bearing	Pressure distribution, friction force, friction coefficient	It is observed that dimple is good for lubricating and minimizes friction force, but there is a loss of load capacity
^37^	CFD	Journal bearing	3D transient flow simulation, load capacity and bearing dynamic coefficient	The CFD results were quite consistent with the experimental results obtained from the test rotor-bearing system
^38^	FEM	Gas journal bearing	Rotation speed, eccentricity ratio and supply pressure	Increasing the eccentricity ratio, supply pressure, and rotation speed at a small average gas film thickness can help improve load capacity and stiffness. The most effective way of reducing attitude angle is to increase supply pressure

Furthermore, the viscosity of the lubricant used has a significant impact on the steady-state performance of hydrodynamic journal bearings. Nanofluids have higher viscosity than conventional fluids in the absence of nanoparticle additions. Nanofluids’ effective viscosity is determined by the concentration and size of nanoparticles. Many classical models of nanofluid viscosities have been constructed in Refs.^39–47^. There is also a scarcity of published data on how nanoparticle lubricant additives affect the dynamic responsiveness and stability of hydrodynamic lubrication. Many researchers investigated the steady-state characteristics of various hydrodynamic journal bearings operating with different nanoparticles as lubricant additives^48–52^. Several prior studies have investigated the characteristics of journal bearings lubricated by couple stress fluids^53–60^. They determined that friction causes a slight rise in lubricant temperature. There has been no mention of using nanolubricants to investigate bearing performance in advanced shapes. The current technology allows for the precise machining of extremely complex shapes. The effects of TiO_2_ nanoparticle volume fraction and nanoparticle aggregate sizes on the steady state characteristics and stability limitations of plain journal bearings were investigated by Awad et al.^61^.

The current work is a continuation of Awad et al.^61^’s analysis to investigate the combined effects of axial changes in bearing geometrical shape and the use of nanoparticle additives with aggregation properties on bearing performance while keeping the radial clearance constant.

## Materials and methods

### Formulation

**Fig. 1:**
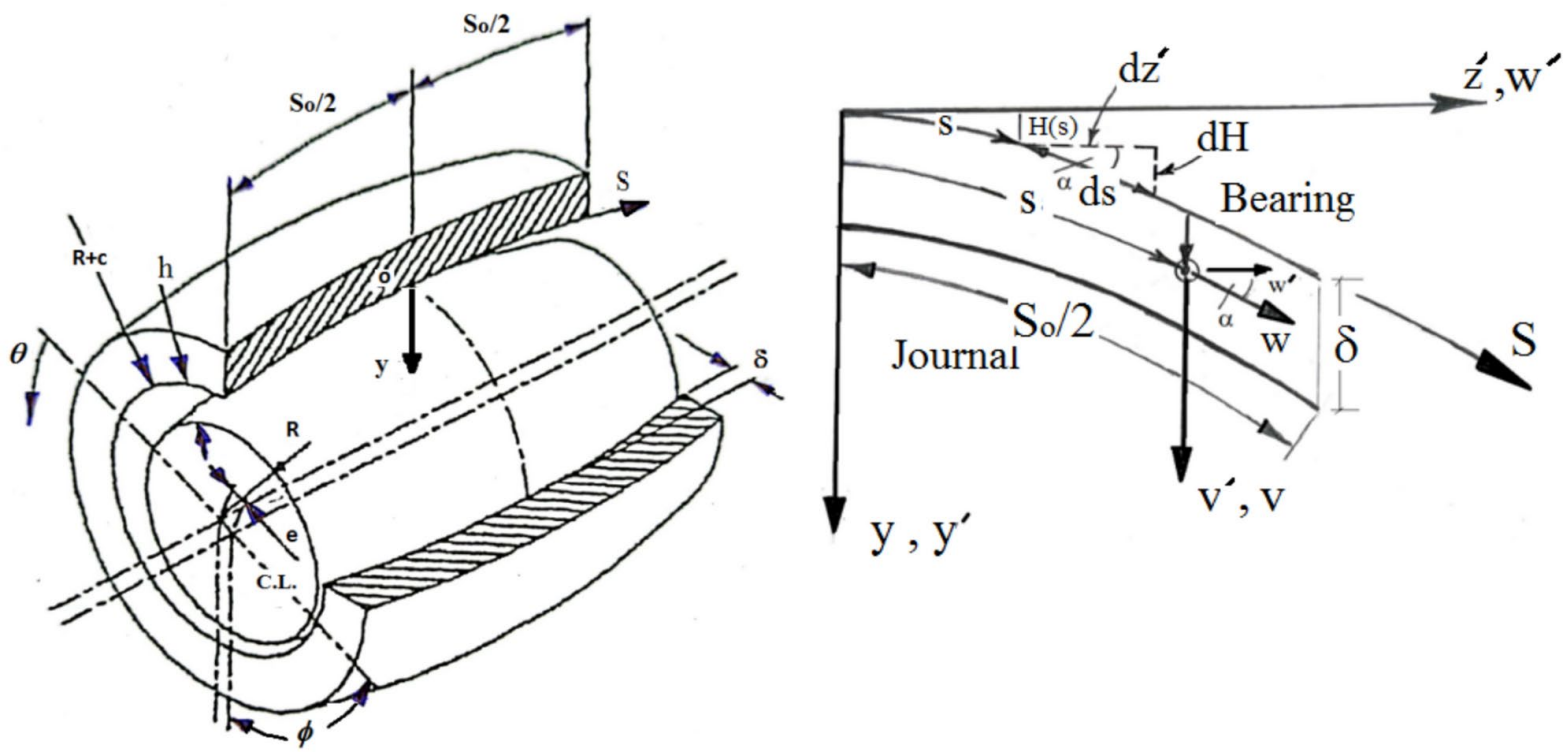
Bearing geometry and curvilinear coordinate system.

**Fig. 2:**
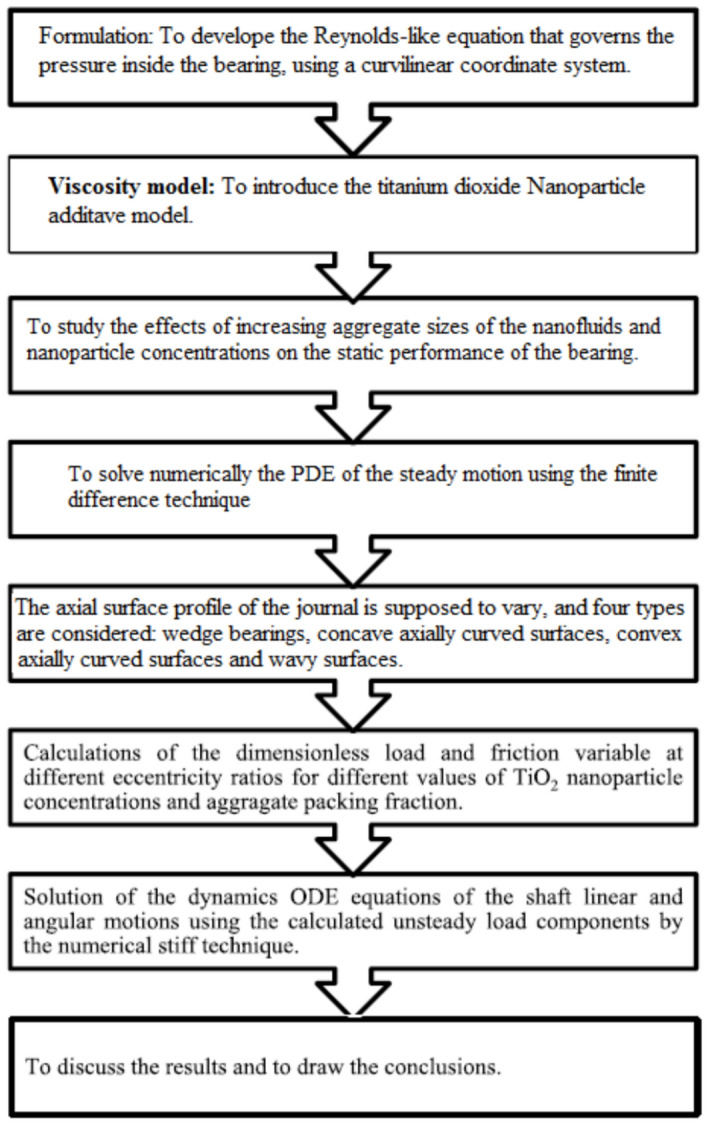
Steps of research methodology.

Figure 1 depicts the bearing arrangement and the suggested curvilinear coordinate system. When the Reynolds number is low, the fluid inertia forces may be ignored compared to the viscous forces, as is always the case with bearing problems. The research methodology used in the current study is depicted in Fig. 2, where a suggested work’s block diagram is presented in order to clarify and simplify the work description.

The equations governing the lubricant flow in the bearing, assuming that the flow is steady, laminar, and incompressible, are thus stated in Cartesian coordinates as,

1 $$\left. \begin{gathered} 0 = - \,\,\frac{1}{\rho }\,\frac{\partial \,p}{{\partial \,x}} + \nu \,\left( {\frac{{\partial^{2} \,u}}{{\partial \,x^{2} }} + \frac{{\partial^{2} \,u}}{{\partial \,y^{{\prime}{2}} }} + \frac{{\partial^{2} \,u}}{{\partial \,z^{{\prime}{2}} }}} \right)\, \hfill \\ 0 = \frac{\partial \,p}{{\partial \,y^{\prime}}} \hfill \\ 0 = - \,\,\frac{1}{\rho }\,\frac{\partial \,p}{{\partial \,z^{\prime}}} + \nu \,\left( {\frac{{\partial^{2} \,w^{\prime}}}{{\partial \,x^{2} }} + \frac{{\partial^{2} \,w^{\prime}}}{{\partial \,y^{{\prime}{2}} }} + \frac{{\partial^{2} \,w^{\prime}}}{{\partial \,z^{{\prime}{2}} }}} \right) \hfill \\ and \hfill \\ 0 = \,\frac{\partial \,u}{{\partial \,x}} + \frac{{\partial \,v^{\prime}}}{{\partial \,y^{\prime}}} + \frac{{\partial \,w^{\prime}}}{{\partial \,z^{\prime}}}\, \hfill \\ \end{gathered} \right\}$$

In terms of the curvilinear coordinates employed, we may write:

2 $$\left. \begin{gathered} \frac{\partial }{{\partial \,y^{\prime}}} = \frac{\partial }{\partial \,y}\,\,and\,\,\frac{{\partial^{2} }}{{\partial \,y^{{\prime}{2}} }} = \frac{{\partial^{2} }}{{\partial \,y^{2} }} \hfill \\ \frac{\partial }{{\partial \,z^{\prime}}} = \frac{1}{E}\left( {\frac{\partial }{\partial \,s} - \dot{H}\frac{\partial }{\partial \,y}} \right) \hfill \\ \frac{{\partial^{2} }}{{\partial \,z^{{\prime}{2}} }} = \frac{1}{{E^{2} }}\left[ {\left( {\dot{H}\frac{{\dot{E}}}{E} - \ddot{H}} \right)\frac{\partial }{\partial \,y}\, - \left( {\frac{{\dot{E}}}{E}} \right)\frac{\partial }{\partial \,s} + \frac{{\partial^{2} }}{{\partial \,s^{2} }} + \dot{H}^{2} \frac{{\partial^{2} }}{{\partial \,y^{2} }} - 2\,\dot{H}\frac{{\partial^{2} }}{\partial \,y\,\partial s}} \right] \hfill \\ \end{gathered} \right\}$$

With $y^{\prime} = y + H$, where $H = H(s)$ is a function that defines the geometrical configuration of the bearing (see Fig. 1) and must be supplied prior to any numerical computations. $E = E(s) = \sqrt {1 - \dot{H}^{2} }$, the dot denotes differentiation with respect to s. Referring to Fig. 1, we may also write, $w^{\prime} = E\,w\,\,and\,\,v^{\prime} = v + \dot{H}\,w$.

Using the relationships (2), Eqns. (1) can be expressed as follows:

3 $$\left. \begin{aligned} 0 = & - \frac{1}{\rho }\,\frac{\partial \,p}{{\partial \,x}} + \nu \,\left[ {\frac{{\partial^{2} \,u}}{{\partial \,x^{2} }} + \left( {1 + \frac{{\dot{H}^{2} }}{{E^{2} }}} \right)\,\frac{{\partial^{2} \,u}}{{\partial \,y^{2} }} + \frac{1}{{E^{2} }}\frac{{\partial^{2} \,u}}{{\partial \,s^{2} }} - \frac{{\dot{E}}}{{E^{3} }}\frac{\partial \,u}{{\partial \,s}}} \right. \\ & \left. { + \frac{1}{{E^{2} }}\left( {\dot{H}\frac{{\dot{E}}}{E} - \ddot{H}} \right)\frac{\partial \,u}{{\partial \,y}} - 2\frac{{\dot{H}}}{{E^{2} }}\frac{{\partial^{2} \,u}}{\partial y\,\partial s}} \right] & & & & & (a) \\ 0 = & \frac{\partial \,p}{{\partial \,y}} & & & & & \;\;\;\;\;\;\;\;(b) \\ 0 = & - \frac{1}{{\rho \,E^{2} }}\,\frac{\partial \,p}{{\partial \,s}} + \nu \,\left[ {\frac{{\partial^{2} \,w}}{{\partial \,x^{2} }} + \left( {1 + \frac{{\dot{H}^{2} }}{{E^{2} }}} \right)\,\frac{{\partial^{2} \,w}}{{\partial \,y^{2} }} + \frac{1}{{E^{2} }}\frac{{\partial^{2} \,w}}{{\partial \,s^{2} }} + \frac{{\dot{E}}}{E}\frac{\partial \,w}{{\partial \,s}}} \right. \\ & + \frac{1}{{E^{2} }}\frac{{\partial^{2} \,w}}{{\partial \,s^{2} }}\left. { - \frac{1}{{E^{2} }}\left( {\dot{H}\frac{{\dot{E}}}{E} + \ddot{H}} \right)\frac{\partial \,w}{{\partial \,y}} - 2\frac{{\dot{H}}}{{E^{2} }}\frac{{\partial^{2} \,w}}{\partial y\,\partial s} + \frac{1}{{E^{3} }}\left( {\ddot{E} - \frac{{\dot{E}^{2} }}{E}} \right)\,w} \right] & & & & & (c) \\ and \\ 0 = & \frac{\partial \,u}{{\partial \,x}} + \frac{\partial \,v}{{\partial \,y}} + \frac{\partial \,w}{{\partial \,s}} + \frac{{\dot{E}}}{E}\,w & & & & & (d) \\ \end{aligned} \right\}$$

Dimensionless quantities are introduced, such as:

4 $$\begin{gathered} \overline{y} = \frac{y}{c}\,\,,\,\,\theta = \frac{x}{R}\,\,,\,\,\overline{s} = \frac{s}{{S_{o} }}\,\,,\,\,\overline{H} = \frac{H}{{S_{o} }}\,\,,\,\,\overline{u} = \frac{u}{\omega \,R}\,\,,\,\,\overline{v} = \frac{v}{\omega \,R}\,\,,\,\,\overline{w} = \frac{w}{\omega \,R}\,\,,\,\,\Delta = \frac{\delta }{{S_{o} }}\,\, \hfill \\ \,and\,\,\overline{p} = \frac{{p\,\,c^{2} }}{{\mu_{bf} \,\omega \,R^{2} }} \hfill \\ \end{gathered}$$

Using the supplied dimensionless variables in (4) and assuming that ${c \mathord{\left/ {\vphantom {c {R < < 1}}} \right. \kern-0pt} {R < < 1}}\,\,and\,\,{c \mathord{\left/ {\vphantom {c {S_{o} < < 1}}} \right. \kern-0pt} {S_{o} < < 1}}$, Eq. (3) can be reduced to,

5 $$\left. \begin{aligned} 0 = & - \,\frac{{\partial \,\overline{p}}}{\partial \,\theta } + \frac{1}{{E^{2} }}\,\frac{{\partial^{2} \,\overline{u}}}{{\partial \,\overline{y}^{2} }} & & (a) \\ 0 = & \frac{{\partial \,\overline{p}}}{{\partial \,\overline{y}}} & & (b) \\ 0 = & - \left( {\frac{R}{{S_{o} }}} \right)\,\frac{{\partial \,\overline{p}}}{{\partial \,\overline{s}}} + \frac{{\partial^{2} \,\overline{w}}}{{\partial \,\overline{y}^{2} }} & & (c) \\ and \\ 0 = & \frac{{\partial \,\overline{u}}}{\partial \,\theta } + \left( \frac{R}{c} \right)\,\frac{{\partial \,\overline{v}}}{{\partial \,\overline{y}}} + \left( {\frac{R}{{S_{o} }}} \right)\,\left( {\frac{{\partial \,\overline{w}}}{{\partial \,\overline{s}}} + \frac{1}{E}\frac{d\,E}{{d\,\overline{s}}}\,\overline{w}} \right) & & (d) \\ \end{aligned} \right\}$$

The velocity components boundary conditions are as follows:

6 $$\left. \begin{aligned} At\,\,\overline{y} = 0: &\,\,\,\, \overline{u}\,\left( {\theta \,,\,\,\overline{s}\,,\,\,0} \right) = 0 \\ & \,\,\,\,\overline{w}\,\left( {\theta \,,\,\,\overline{s}\,,\,\,0} \right) = 0 \\ At\,\,\overline{y} = h^{*} : & \,\,\,\,\overline{u}\,\left( {\theta \,,\,\,\overline{s}\,,\,\,h^{*} } \right) = 1 + \frac{{S_{o} }}{R}\,\left( {\Delta - \overline{H}} \right) \\ & \,\,\,\,\overline{w}\,\left( {\theta \,,\,\,\overline{s}\,,\,\,h^{*} } \right) = 0 \\ \end{aligned} \right\}$$

where $h^{*}$ is the dimensionless film thickness, $h^{*} = {h \mathord{\left/ {\vphantom {h c}} \right. \kern-0pt} c} = 1 + \varepsilon \,\,\cos \,\,(\theta )$, and the dimensionless maximum variation of the geometry is defined as $\Delta = {\delta \mathord{\left/ {\vphantom {\delta {S_{o} }}} \right. \kern-0pt} {S_{o} }}$.

The velocity field can be obtained by integrating Eqs. (5a and c) twice with respect to $\overline{y}$ and applying the boundary conditions at $\overline{y} = 0\,\,and\,\,\overline{y} = h^{*}$. The velocity components and velocity gradients are,

7a $$\left. \begin{gathered} \overline{u} = \frac{{E^{2} }}{2}\left( {\overline{y}^{2} - \overline{y}\,h^{*} } \right)\,\frac{{\partial \,\overline{p}}}{\partial \,\theta } + \frac{{\overline{y}}}{{h^{*} }}\,\left[ {1 + \frac{{S_{o} }}{R}\,\left( {\Delta - \overline{H}} \right)} \right] \hfill \\ \overline{w} = \frac{1}{2}\left( {\frac{R}{{S_{o} }}} \right)\,\left( {\overline{y}^{2} - \overline{y}\,h^{*} } \right)\,\frac{{\partial \,\overline{p}}}{{\partial \,\overline{s}}} \hfill \\ \end{gathered} \right\}$$

7b $$\left. \begin{gathered} \frac{{\partial \,\overline{u}}}{{\partial \,\overline{y}}} = \,E^{2} \left( {\overline{y} - \frac{{h^{*} }}{2}} \right)\,\frac{{\partial \,\overline{p}}}{\partial \,\theta } + \frac{1}{{h^{*} }}\,\left[ {1 + \frac{{S_{o} }}{R}\,\left( {\Delta - \overline{H}} \right)} \right] \hfill \\ \frac{{\partial \,\overline{w}}}{{\partial \,\overline{y}}} = \left( {\frac{R}{{S_{o} }}} \right)\,\left( {\overline{y} - \frac{{h^{*} }}{2}} \right)\,\frac{{\partial \,\overline{p}}}{{\partial \,\overline{s}}} \hfill \\ \end{gathered} \right\}$$

Integrating Eq. (5d) with respect to $\overline{y}$ across the film yields,

8 $$\frac{\partial }{\partial \,\theta }\left( {E\,h^{{*^{3} }} \frac{{\partial \,\overline{p}}}{\partial \,\theta }} \right) + \left( {\frac{R}{{S_{o} \,E}}} \right)^{2} \,\frac{\partial }{{\partial \,\overline{s}}}\left( {E\,h^{{*^{3} }} \frac{{\partial \,\overline{p}}}{{\partial \,\overline{s}}}} \right) = \frac{6}{E}\,\left[ {1 + \frac{{S_{o} }}{R}\left( {\Delta - \overline{H}} \right)} \right]\,\frac{{\partial \,h^{*} }}{\partial \,\theta } + \frac{12}{E}\frac{{\partial \,h^{*} }}{{\partial \,\overline{t}}}$$

Equation (8) is a general form of Reynolds equation that governs the pressure inside a bearing with a variable axial shape. Axially, the radial clearance is always kept constant. Assuming steady state film operation, that is ${{\partial \,h^{*} } \mathord{\left/ {\vphantom {{\partial \,h^{*} } {\partial \,\mathop t\limits^{\_} = 0}}} \right. \kern-0pt} {\partial \,\mathop t\limits^{\_} = 0}}$,and substituting ${{\partial \,h^{*} } \mathord{\left/ {\vphantom {{\partial \,h^{*} } {\partial \,\theta = - \varepsilon \,\sin \,(\theta )}}} \right. \kern-0pt} {\partial \,\theta = - \varepsilon \,\sin \,(\theta )}}$ into Eq. (8) we have,

9 $$\frac{\partial }{\partial \,\theta }\left( {E\,h^{{*^{3} }} \frac{{\partial \,\overline{p}}}{\partial \,\theta }} \right) + \left( {\frac{R}{{S_{o} \,E}}} \right)^{2} \,\frac{\partial }{{\partial \,\overline{s}}}\left( {E\,h^{{*^{3} }} \frac{{\partial \,\overline{p}}}{{\partial \,\overline{s}}}} \right) = - \,\left( {\frac{6\,\varepsilon }{E}} \right)\,\left[ {1 + \frac{{S_{o} }}{R}\left( {\Delta - \overline{H}} \right)} \right]\,\,\sin \,(\theta )$$

The boundary conditions for the pressure variable $\overline{p}$ are,

10 $$\left. \begin{gathered} \overline{p}\,\left( {0\,,\,\,\overline{s}} \right) = 0\,\,,\,\,\,\,\overline{p}\,\left( {\theta_{eff} \,,\,\overline{s}} \right) = \left. {\frac{{\partial \,\overline{p}}}{\partial \,\theta }} \right|_{{\theta_{eff} }} = 0 \hfill \\ \frac{{\partial \,\overline{p}\,(\theta \,,\,\,0)}}{{\partial \,\overline{s}}} = 0\,,\,\,\overline{p}\,\left( {\theta \,,\,\,{1 \mathord{\left/ {\vphantom {1 2}} \right. \kern-0pt} 2}} \right) = 0 \hfill \\ \end{gathered} \right\}$$

In a dimensionless form, the radial and tangential load components can be represented as,

11 $$\left. \begin{gathered} \overline{W}_{r} = \frac{{W_{r} }}{{\left( {\frac{{\mu_{bf} \,\omega \,R^{3} S_{o} }}{{c^{2} }}} \right)}} = - \,2\,\,\int\limits_{0}^{{{1 \mathord{\left/ {\vphantom {1 2}} \right. \kern-0pt} 2}}} {} \int\limits_{0}^{{\theta_{eff} }} {E\,\overline{p}} \left[ {1 + \left( {\frac{{S_{o} }}{R}} \right)\,\left( {\Delta - \overline{H}} \right)} \right]\,\,\cos \,(\theta )\,\,d\theta \,d\,\overline{s} \hfill \\ \overline{W}_{t} = \frac{{W_{t} }}{{\left( {\frac{{\mu_{bf} \,\omega \,R^{3} S_{o} }}{{c^{2} }}} \right)}} = \,\,\,2\,\,\int\limits_{0}^{{{1 \mathord{\left/ {\vphantom {1 2}} \right. \kern-0pt} 2}}} {} \int\limits_{0}^{{\theta_{eff} }} {E\,\overline{p}} \left[ {1 + \left( {\frac{{S_{o} }}{R}} \right)\,\left( {\Delta - \overline{H}} \right)} \right]\,\,\,\sin \,(\theta )\,\,d\theta \,d\,\overline{s} \hfill \\ \end{gathered} \right\}$$

The dimensionless resultant load $\overline{W}$ and the attitude angle $\phi$ may be calculated from,

$$\overline{W} = \left( {\overline{W}_{r}^{2} + \overline{W}_{t}^{2} } \right)^{{{1 \mathord{\left/ {\vphantom {1 2}} \right. \kern-0pt} 2}}} \,\,,\,\,and\,\,\phi = \tan^{ - 1} \left( {\frac{{\overline{W}_{t} }}{{\overline{W}_{r} }}} \right)$$

Integrating the shear stress around the journal surface yields the friction force, which may be expressed in dimensionless form as,

12 $$\left. \begin{gathered} \overline{F}_{f} = \frac{{F_{f} }}{{\left( {\frac{{\mu_{bf} \,\omega \,R^{2} S_{o} }}{c}} \right)}} = \,2\,\,\int\limits_{0}^{{{1 \mathord{\left/ {\vphantom {1 2}} \right. \kern-0pt} 2}}} {} \int\limits_{0}^{2\,\pi } {E\,\,\left. {\frac{{\partial \,\overline{u}}}{{\partial \,\overline{y}}}} \right|_{{\overline{y} = h^{*} }} \,} \left[ {1 + \left( {\frac{{S_{o} }}{R}} \right)\,\left( {\Delta - \overline{H}} \right)} \right]\,\,\,d\theta \,d\,\overline{s} \hfill \\ where\,\,\left. {\frac{{\partial \,\overline{u}}}{{\partial \,\overline{y}}}} \right|_{{\overline{y} = h^{*} }} = \frac{{E^{2} }}{2}\,h^{*} \frac{{\partial \,\overline{p}}}{\partial \,\theta } + \left( {\frac{1}{{h^{*} }}} \right)\,\left[ {1 + \left( {\frac{{S_{o} }}{R}} \right)\,\left( {\Delta - \overline{H}} \right)} \right] \hfill \\ \end{gathered} \right\}$$

In dimensionless form, the force acting normal to the journal surface is to be calculated from,

13 $$\overline{F}_{n} = \frac{{F_{n} }}{{\left( {\frac{{\mu_{bf} \,\omega \,R^{3} S_{o} }}{{c^{2} }}} \right)}} = \left( {\overline{F}_{{n_{r} }}^{2} + \overline{F}_{{n_{t} }}^{2} } \right)^{{{1 \mathord{\left/ {\vphantom {1 2}} \right. \kern-0pt} 2}}}$$

where $$\overline{F}_{{n_{r} }}^{{}} = \frac{{F_{{n_{r} }} }}{{\left( {\frac{{\mu_{bf} \,\omega \,R^{3} S_{o} }}{{c^{2} }}} \right)}} = - \,2\,\,\int\limits_{0}^{{{1 \mathord{\left/ {\vphantom {1 2}} \right. \kern-0pt} 2}}} {} \int\limits_{0}^{{\theta_{eff} }} {\,\overline{p}} \,\cos \,(\theta )\,\left[ {1 + \left( {\frac{{S_{o} }}{R}} \right)\,\left( {\Delta - \overline{H}} \right)} \right]\,\,\,d\theta \,d\,\overline{s}$$.

And $$\overline{F}_{{n_{t} }} = \frac{{F_{{n_{t} }} }}{{\left( {\frac{{\mu_{bf} \,\omega \,R^{3} S_{o} }}{{c^{2} }}} \right)}} = \,\,\,2\,\,\int\limits_{0}^{{{1 \mathord{\left/ {\vphantom {1 2}} \right. \kern-0pt} 2}}} {} \int\limits_{0}^{{\theta_{eff} }} {\,\overline{p}\,\sin \,(\theta )\,} \left[ {1 + \left( {\frac{{S_{o} }}{R}} \right)\,\left( {\Delta - \overline{H}} \right)} \right]\,\,\,d\theta \,d\,\overline{s}$$.

**Fig. 3:**
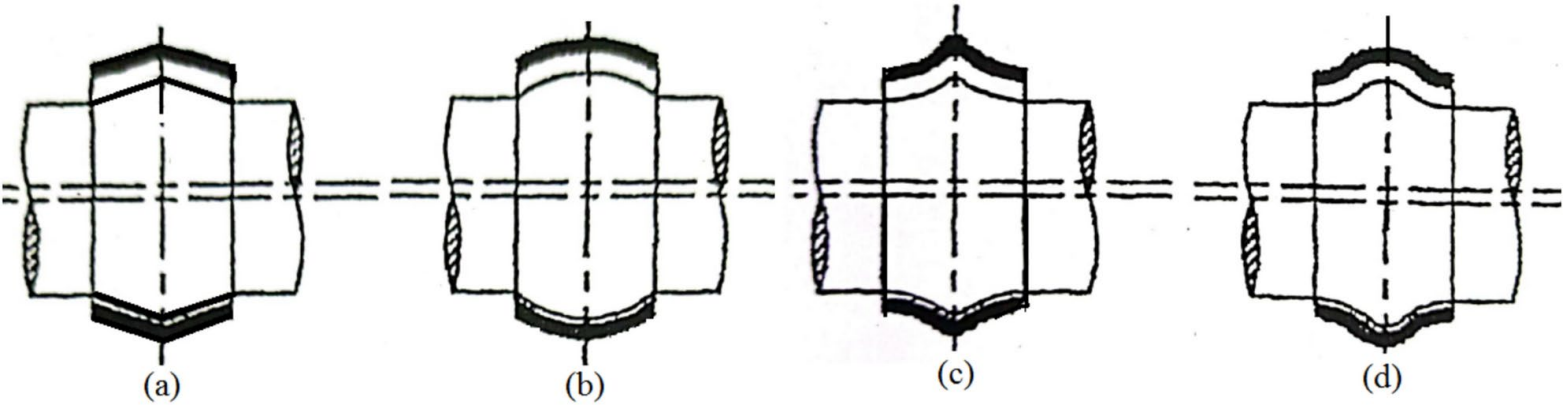
Selected bearing geometrical configurations. (**a**) Wedge; (**b**) Concave; (**c**) Convex; (**d**) Wavy.

The friction parameter (variable) $C_{f} = f\,\left( {{R \mathord{\left/ {\vphantom {R c}} \right. \kern-0pt} c}} \right)$ may be calculated from $f\,\left( {{R \mathord{\left/ {\vphantom {R c}} \right. \kern-0pt} c}} \right) = {{\overline{F}_{f} } \mathord{\left/ {\vphantom {{\overline{F}_{f} } {\overline{F}_{n} }}} \right. \kern-0pt} {\overline{F}_{n} }}$, where $f\, = {{F_{f} } \mathord{\left/ {\vphantom {{F_{f} } {F_{n} }}} \right. \kern-0pt} {F_{n} }}$_._ In Fig. 3, four geometrical bearing configurations are considered for comparison with the plain cylindrical bearing.

The axial surface profile of the journal is supposed to vary, and four types are considered:

14 $$\left. {\begin{array}{*{20}l} {Wedge\,\,bearing:} \hfill & {\overline{H} = 2 \Delta\,\,\overline{s}} \hfill \\ {Concave\,\,axially\,\,curved\,\,surfaces:} \hfill & {\overline{H} = 4{\mkern 1mu} \Delta {\mkern 1mu} {\mkern 1mu} \overline{s}^{2} } \hfill \\ {Convex\,\,axially\,\,curved\,\,surfaces:} \hfill & {\overline{H} = 4{\mkern 1mu} \Delta {\mkern 1mu} {\mkern 1mu} \left( {1 - \overline{s}} \right){\mkern 1mu} \overline{s}} \hfill \\ {Wavy\,\,surfaces:} \hfill & {\overline{H} = \frac{\Delta }{2}{\mkern 1mu} \left( {1 - \cos ({\mkern 1mu} 2{\mkern 1mu} \pi {\mkern 1mu} \overline{s}{\mkern 1mu} )} \right)} \hfill \\ \end{array} } \right\}$$

The coordinate $\overline{s}$ can be linked to the coordinate $\overline{z} = {{z^{\prime}} \mathord{\left/ {\vphantom {{z^{\prime}} {S_{o} }}} \right. \kern-0pt} {S_{o} }}$ by,

$$\overline{z} = \int\limits_{0}^{{\overline{s}}} {\sqrt {1 - \left( {{{d\,\overline{H}} \mathord{\left/ {\vphantom {{d\,\overline{H}} {d\,\overline{s}}}} \right. \kern-0pt} {d\,\overline{s}}}} \right)^{2} } \,d\,\overline{s} = \int\limits_{0}^{{\overline{s}}} {E\,d\,\overline{s}} }$$.

The common parameter for the geometries chosen is the greatest variation of the geometry $\overline{H}_{\max } = \Delta$, which should be practically <  < 1. In this paper, computations are conducted for $\Delta = 0.01\,,\,\,0.05\,,\,\,0.1\,,\,\,and\,\,0.15$.

### Viscosity model $\left( {\overline{\mu }} \right)$

In the present study, the updated Krieger–Dougherty viscosity model may be used as^19^,

15 $$\overline{\mu } = \frac{{\mu_{nf} }}{{\mu_{bl} }} = \left( {1 - \frac{{\Phi_{a} }}{{\Phi_{m} }}} \right)^{{\, - 2.5\,\Phi_{m} }}$$

With $$\Phi_{a} = \Phi \,\left( {\frac{{a_{a} }}{a}} \right)^{{3 - D^{*} }}$$

Where,$a_{a} \,\,and\,\,a$, are the radii of aggregates and primary particles, D^*^ is the fractional index, which has a standard value of 1.8 for nanofluids^44^. $\Phi_{m}$ is the maximum particle packing fraction, $\Phi_{m} = 0.605$ at high shear stress rates^44^. Using the reported values of D^*^ and $\Phi_{m}$, the modified Kriegerr-Dougherty equation may be written as,

16 $$\overline{\mu } = \frac{{\mu_{nf} }}{{\mu_{bl} }} = \left( {1 - \frac{\Phi }{{\Phi_{m} }}\,\left( \beta \right)^{\,1.2} } \right)^{{\, - 2.5\,\Phi_{m} }}$$

where $\beta = \left( {{{a_{a} } \mathord{\left/ {\vphantom {{a_{a} } a}} \right. \kern-0pt} a}} \right)$ is the aggregate packing fraction, which depends on the type of the nanoparticles and their sizes. Binu et al.^50^ carried out experimental measurements on TiO_2_ nanoparticles of $a < 100\,{\text{nm}}$ and distributed in SAE30 engine oil at different volume fractions ranging from $\Phi = 0.0001\,\,to\,\,0.005$. They used the DLS analysis and found that the mean aggregate particle size $a_{a} = 777\,{\text{nm}}$ and the aggregate packing fraction was estimated to be $\beta = 7.77$. This means that the TiO_2_ nanoparticle aggregates are roughly 7.77 times the primary particle size of 100 nm. They documented that Eq. (16) has a fairly good agreement with the measured viscosities for different values of volume fraction. In the present analysis, for comparison purpose, the study is carried out for volume fraction ranging from 0.001 to 0.01 with various values of aggregate particle packing fraction $\beta = 4\,,\,\,7.77\,\,and\,10$.

### Solution methodology

The Reynolds-like Eq. (9) and boundary conditions (10) were numerically solved with the finite difference technique. To solve the sets of simultaneous equations, a FORTRAN-based computational algorithm^62^ is constructed in order to be used with the successive relaxation method. To consider a non-cavitating model, set all negative pressure variables to zero throughout the solution^61^. The iteration process continues until convergence with a relative tolerance of 0.01 is reached. The modified Krieger–Dougherty model (Eq. 16) is employed in the discretized Reynolds equations to calculate steady-state pressure distributions in the bearing lubricant film. The bearing properties of TiO_2_ nanoparticle volume fractions are studied for different aggregate size values.

## Results and discussions

The steady state journal bearing characteristics is affected significantly by using the journal bearing with an axial variation in geometrical shape as well as the use of nanoparticles as lubricant additives. Four different shapes for the bearing having wedge, concave, convex and wavy surfaces are made. Based on the experimental work of Binu et al.^26^, the modified Krieger–Dougherty model, Eq. 16, is used to calculate the viscosity of the nanolubricant. In the formulation of nanolubricant, TiO_2_ nanoparticles (scale 100 nm) and SAE30 engine oil are employed, with volume fractions ranging from 0.001 to 0.01 at various nanoparticle aggregate sizes. According to the authors’ knowledge, there are no published studies on how nanoparticle concentrations and aggregate particle sizes affect journal bearing performance using axial geometrical variations. In Refs^50,60^, researchers examined the impact of titanium dioxide nanoparticles as lubricant additives on steady-state performance^50,60^ and stability limitations^60^. The current study found a high agreement between the model developed and data from the cited references for a cylindrical bearing operating under steady state conditions. El-Gamal^15^ provided an analysis of the steady state performance of a wedge-shaped hydrodynamic journal bearing, comparing the results of the dimensionless wedged-bearing load produced in the current work to the results obtained in^15^. A good qualitative agreement has been reached. The largest disparity in dimensionless load is 5.26% at eccentricity ratio 0.2, decreasing to 1.125% at eccentricity ratio 0.9.

### Bearing characteristics for different geometries

**Fig. 4:**
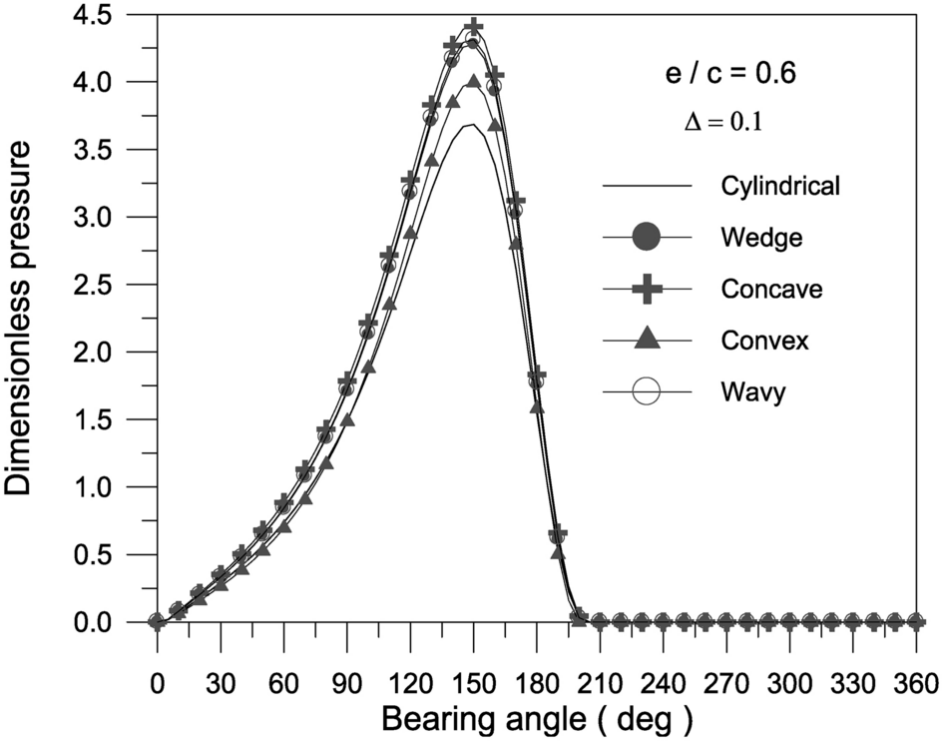
Effect of axial geometrical variations (bearing geometry) on pressure distribution along circumferential direction at bearing mid-plane at ${{\Phi = 0\,\,and\,\,S_{o} } \mathord{\left/ {\vphantom {{\Phi = 0\,\,and\,\,S_{o} } {D = 1}}} \right. \kern-0pt} {D = 1}}$.

**Fig. 5:**
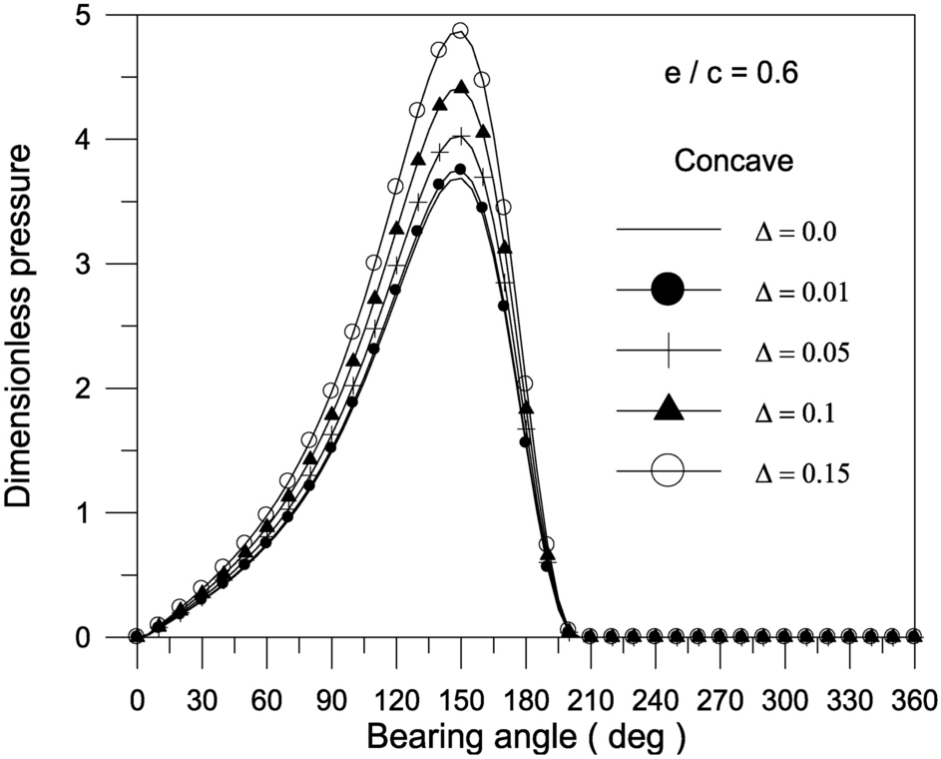
Effect of the maximum axial variation of the concave bearing geometry on pressure distribution along circumferential direction at bearing mid-plane with ${{\Phi = 0\,\,and\,\,S_{o} } \mathord{\left/ {\vphantom {{\Phi = 0\,\,and\,\,S_{o} } {D = 1}}} \right. \kern-0pt} {D = 1}}$.

**Fig. 6:**
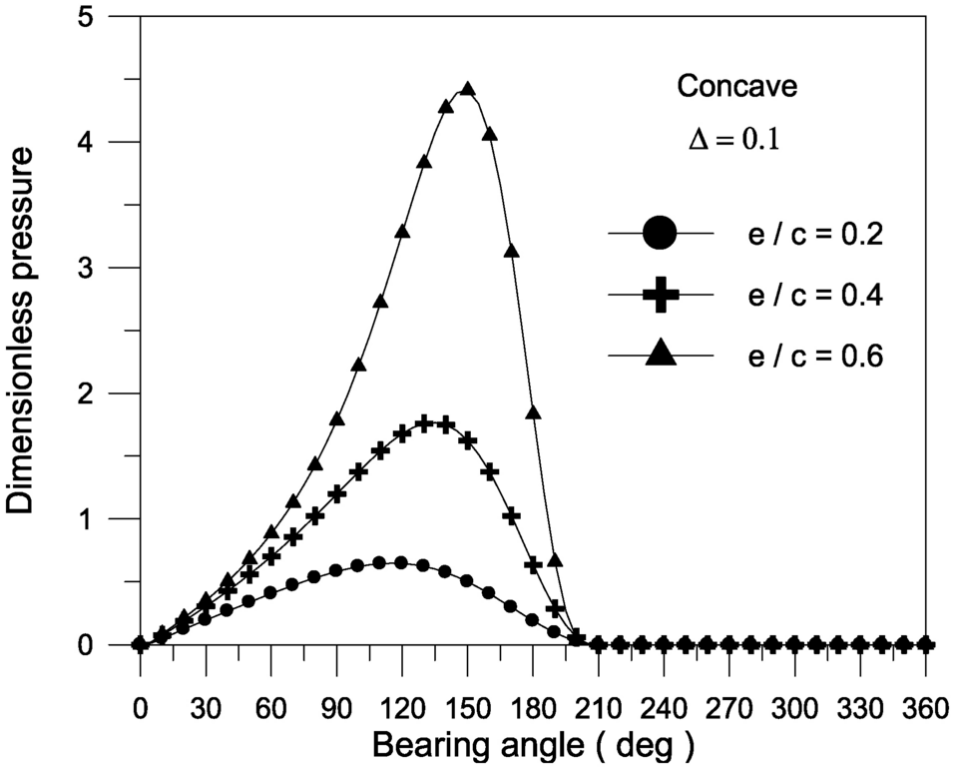
Effect of eccentricity ratio on pressure distribution along circumferential direction at bearing mid-plane for concave axial variation of the bearing geometry at ${{\Phi = 0\,\,and\,\,S_{o} } \mathord{\left/ {\vphantom {{\Phi = 0\,\,and\,\,S_{o} } {D = 1}}} \right. \kern-0pt} {D = 1}}$.

The pressure distribution inside the bearing at the mid-plane is depicted in Figs. 4, 5 and 6.

**Fig. 7:**
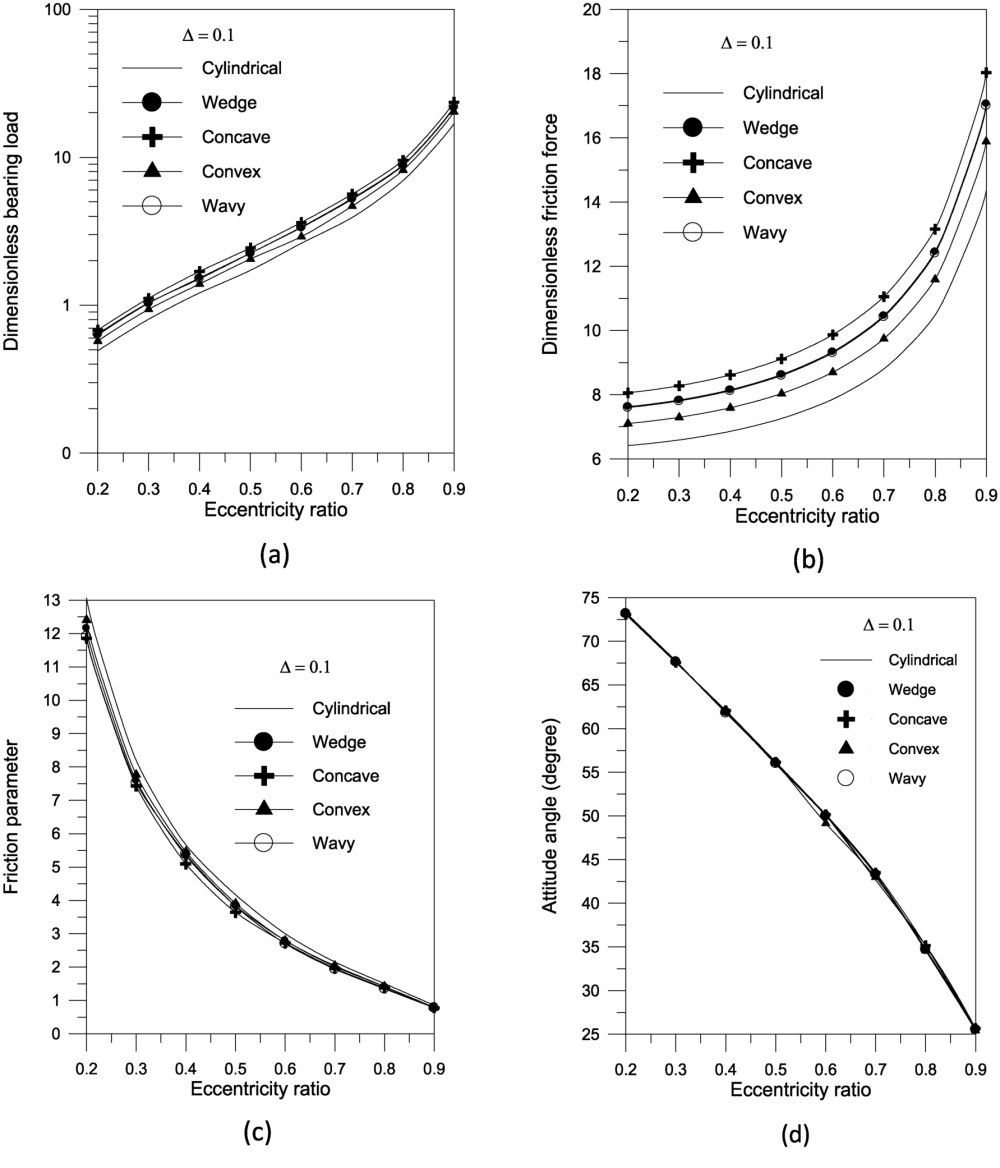
Bearing characteristics (bearing load, friction force, friction parameter and attitude angle) for different geometries with ${{\Phi = 0\,\,and\,\,S_{o} } \mathord{\left/ {\vphantom {{\Phi = 0\,\,and\,\,S_{o} } {D = 1}}} \right. \kern-0pt} {D = 1}}$.

The findings reveal that a concave surface creates higher pressure levels inside the fluid film. The maximum geometric variation raises the pressure distribution values in the bearing oil film. Furthermore, the higher the eccentricity ratio, the higher the pressure values in the bearing fluid film. Figure 7 shows the results of the bearing characteristics $\overline{W}\,,\,\,\overline{F}_{f} \,,\,\,f\,({R \mathord{\left/ {\vphantom {R {c)}}} \right. \kern-0pt} {c)}}$ and $\phi$ obtained for the wedge, concave, convex and wavy shaped geometries selected. The results are presented for $\Delta = 0.1\,\,and\,\,{{S_{o} } \mathord{\left/ {\vphantom {{S_{o} } {D = 1}}} \right. \kern-0pt} {D = 1}}$ with $\Phi = 0$. The plain cylindrical bearing characteristics are also shown in the same Fig. 7a–d for comparison. It is remarkable that all geometries appear to have better load carrying capacity than a plain cylindrical bearing. This is the case at any eccentricity ratio. For all geometries, the load carrying capacity increases with the increase in eccentricity ratio. When the geometries are compared with each other, the concave geometry is found to give the largest load carrying capacity $\overline{W}$ at any eccentricity ratio $\varepsilon$. On the other hand, the convex shape is seen to give the smallest $\overline{W}$ at any $\varepsilon$. Figure 7c shows that the friction parameter $f(R/c)$ is seen to decrease for the geometries considered compared to the plain cylindrical journal bearing at any $\varepsilon$.The geometries considered show a negligible differences in attitude angle when compared to the plain cylindrical bearing at any $\varepsilon$, see Fig. 7d.

**Fig. 8:**
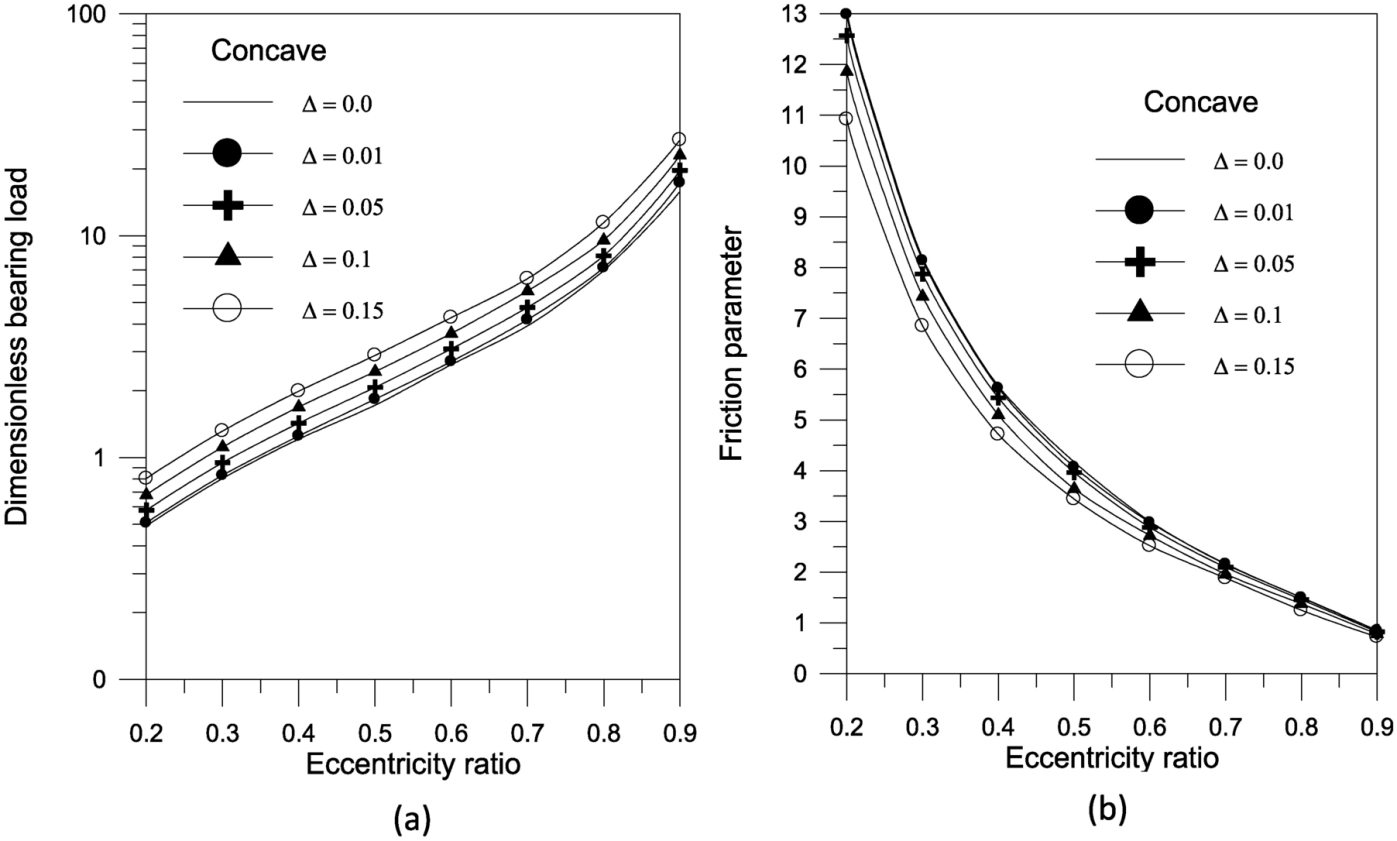
Effect of maximum axial variation on bearing characteristics (bearing load $\overline{W}$ and friction parameter $f\,(R/c)$) for concave bearing geometry at ${{\Phi = 0\,\,and\,\,S_{o} } \mathord{\left/ {\vphantom {{\Phi = 0\,\,and\,\,S_{o} } {D = 1}}} \right. \kern-0pt} {D = 1}}$.

The superiority of concave geometry over other shapes can be seen. The results in Fig. 8a show that increasing the largest variation of the geometry $\Delta$ increases the bearing load capacity for concave axially curved surfaces. Furthermore, as shown in Fig. 8b, the largest change in the concave surface shape shows a significant reduction in the friction parameter at low eccentricity ratios.

### Bearing characteristics using titanium dioxide nanoparticles as lubricant additive

**Fig. 9:**
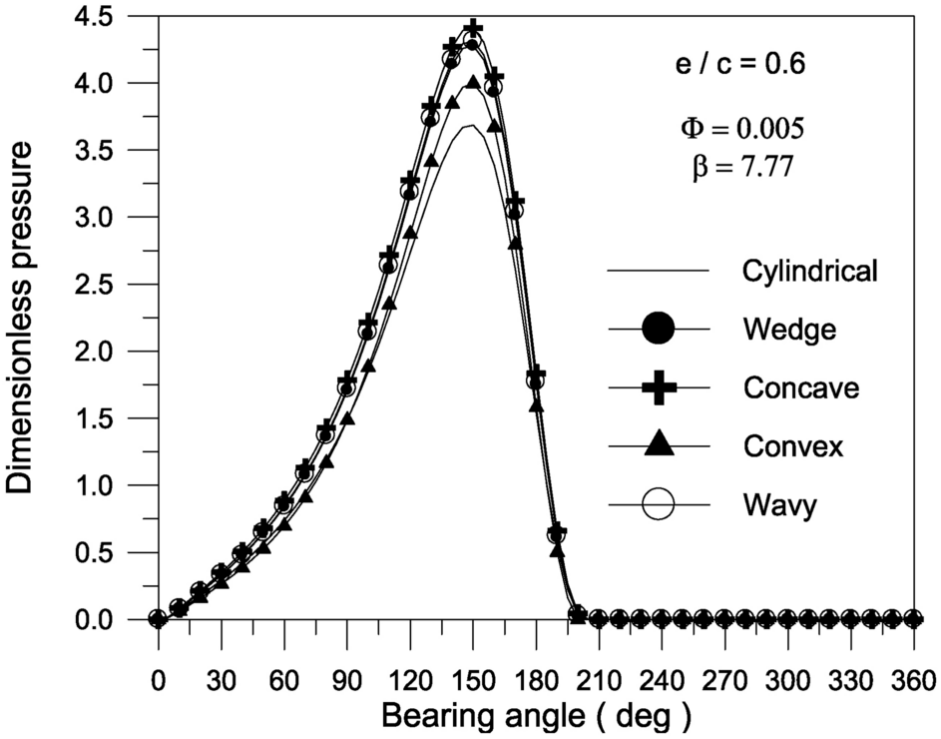
Effect of bearing geometry on pressure distribution along circumferential direction at bearing mid-plane at ${{\Phi = 0.005\,\,,\,\,\beta = 7.77\,\,and\,\,S_{o} } \mathord{\left/ {\vphantom {{\Phi = 0.005\,\,,\,\,\beta = 7.77\,\,and\,\,S_{o} } {D = 1}}} \right. \kern-0pt} {D = 1}}$.

The effect of TiO_2_ nanoparticles with $\Phi = 0.005$ and aggregate packing fraction $\beta = 7.77$ on the pressure distribution in the oil film along the circumferential direction at the bearing mid-plane at $\varepsilon = 0.6$ is shown in Fig. 9. Figure 9 illustrates the pressure distribution along the circumferential direction at bearing mid-plane for all of the axial geometrical variations considered in the present investigation, namely wavy, concave, convex, and wavy. In comparison to the pressure distribution for plain bearings, the presence of any of the aforementioned axial variations causes an increase in the generated pressure inside the oil film. The findings reveal that the concave surface shape causes higher pressure distribution in the oil film than the other surface geometries.

**Fig. 10:**
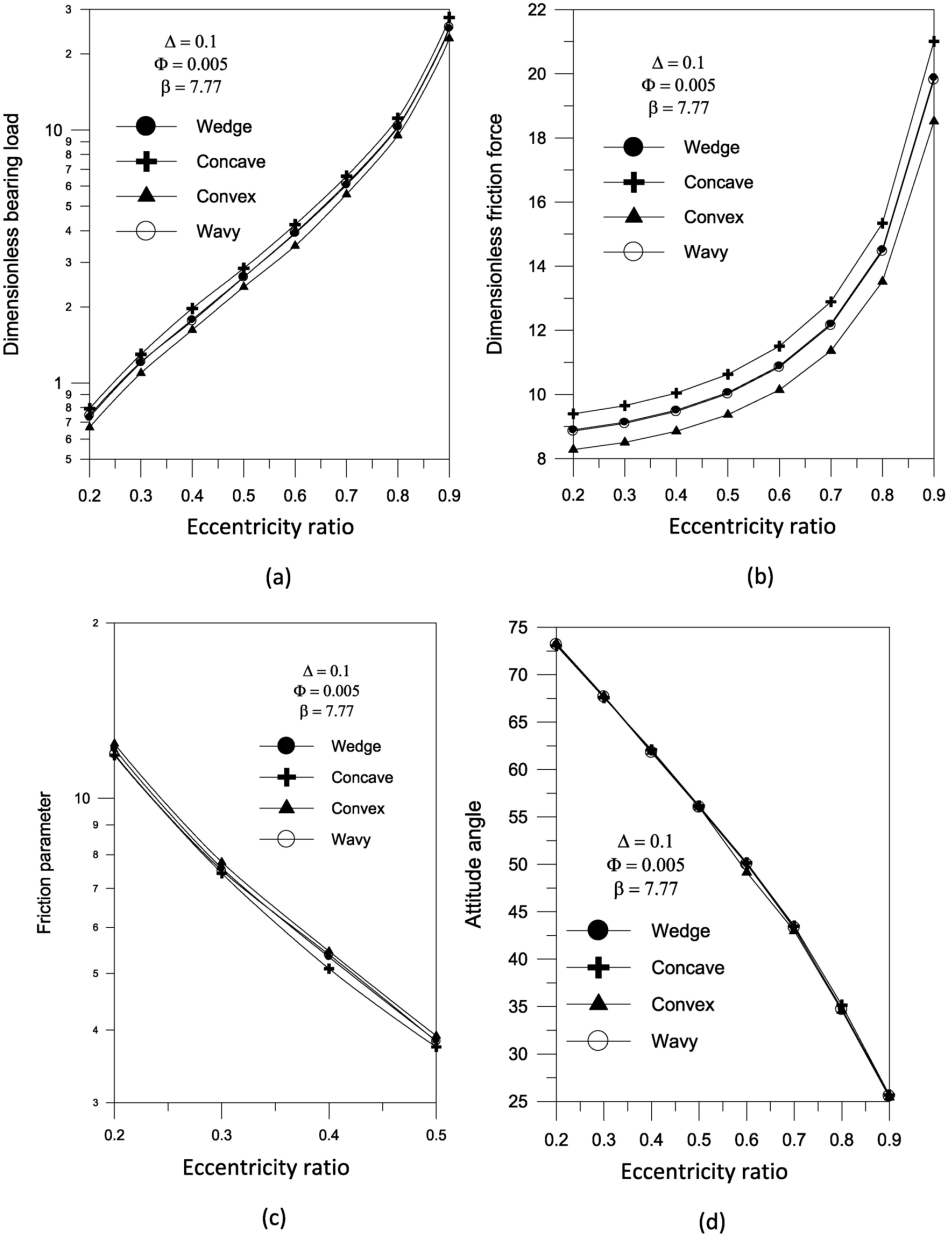
Bearing characteristics (bearing load $\overline{W}$, friction force $\overline{F}_{f}$, friction parameter $f\,(R/c)$ and attitude angle $\phi$) for different geometries with ${{\Phi = 0.005\,\,,\,\,\beta = 7.77\,\,\,and\,\,S_{o} } \mathord{\left/ {\vphantom {{\Phi = 0.005\,\,,\,\,\beta = 7.77\,\,\,and\,\,S_{o} } {D = 1}}} \right. \kern-0pt} {D = 1}}$.

**Fig. 11:**
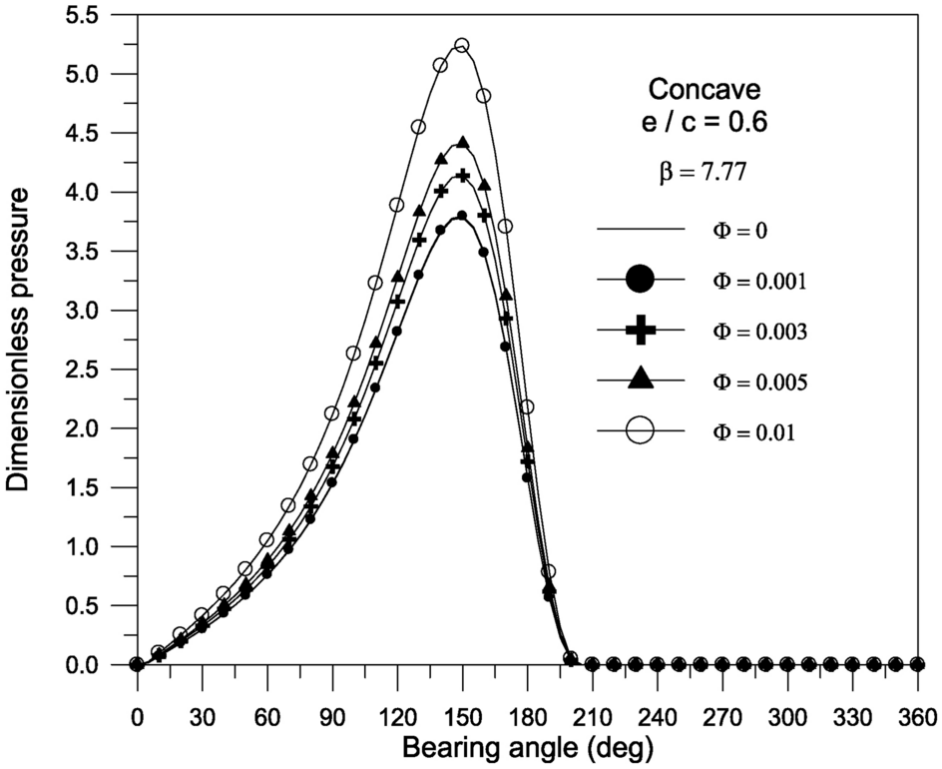
Effect of volume fraction $\Phi$ on pressure distribution along circumferential direction for concave bearing geometry at mid-plane with ${{\,S_{o} } \mathord{\left/ {\vphantom {{\,S_{o} } {D = 1}}} \right. \kern-0pt} {D = 1}}$.

**Fig. 12:**
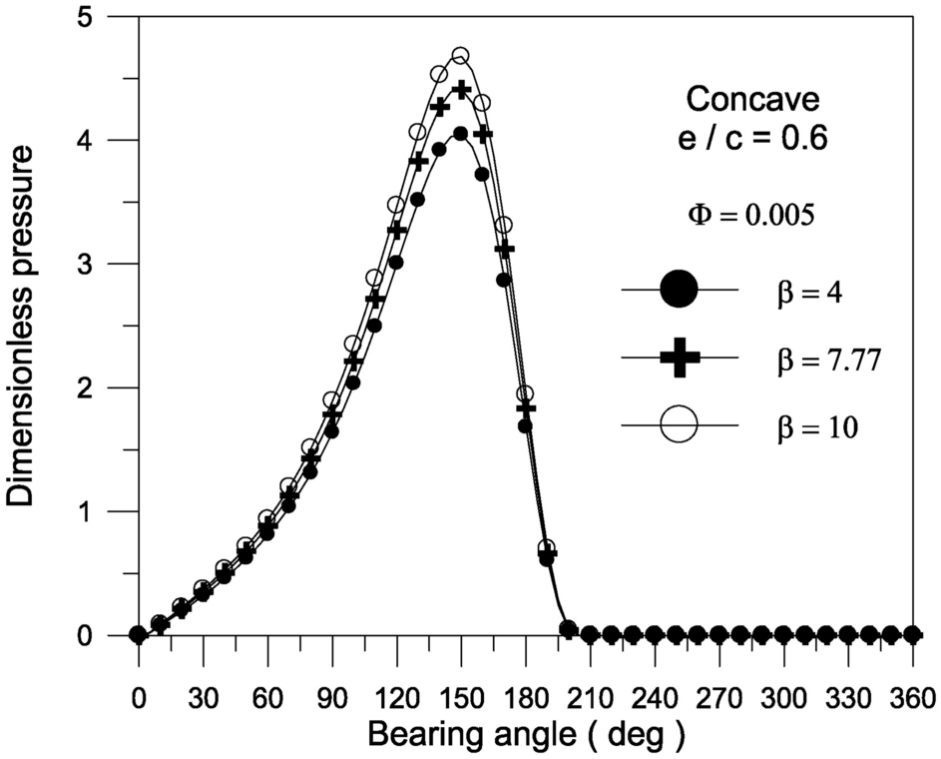
Effect of aggregate packing fraction $\beta$ on pressure distribution along circumferential direction for concave surface bearing geometry at mid-plane with ${{\Phi = 0.005\,\,and\,\,\,S_{o} } \mathord{\left/ {\vphantom {{\Phi = 0.005\,\,and\,\,\,S_{o} } {D = 1}}} \right. \kern-0pt} {D = 1}}$.

Bearing characteristics (bearing load, friction force, friction parameter, and attitude angle) for various geometries using a TiO_2_ lubricant additive with a volume fraction $\Phi = 0.005$ and aggregate packing fraction $\beta = 7.77$ are shown in Fig. 10. The bearing’s behavior is similar to that shown in Fig. 7, and the superiority of concave geometry over other shapes persists. For the concave surface geometry, the effect of TiO_2_ volume fractions; ranging from 0 to 0.01; on the pressure distributions inside the bearing is explored in Fig. 11. It can be seen that as the volume fraction increases, the pressure inside the bearing increases. At higher volume fractions, the rise in pressure is found to be more pronounced. As shown in Fig. 12, the maximum value of the bearing oil film pressure distribution increases as the aggregate packing fraction value (described in viscosity model) increases. With increasing aggregate packing fractions, the pressure distribution becomes more pronounced.

**Fig. 13:**
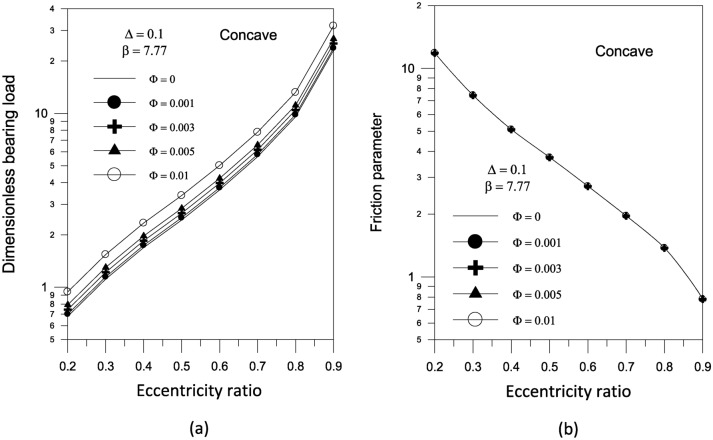
Effect of nanoparticle volume fraction on bearing characteristics (bearing load and friction parameter $f\,(R/c)$) for concave bearing geometry at ${{\Phi = 0.005\,\,,\,\,\beta = 7.77\,\,\,and\,\,S_{o} } \mathord{\left/ {\vphantom {{\Phi = 0.005\,\,,\,\,\beta = 7.77\,\,\,and\,\,S_{o} } {D = 1}}} \right. \kern-0pt} {D = 1}}$.

As demonstrated in Figure 13a, the effect of various TiO_2_ volume fractions on bearing load capacity for concave surface bearing is analyzed and depicted. The findings show that the existence of TiO_2_ nanoparticle lubricant additives increases the bearing load carrying ability. The increase in bearing load is found to be more pronounced at higher TiO_2_ nanoparticle volume fraction values. According to Fig. 13b, increasing the volume fraction has no sensible effect on the bearing friction parameter.

**Fig. 14:**
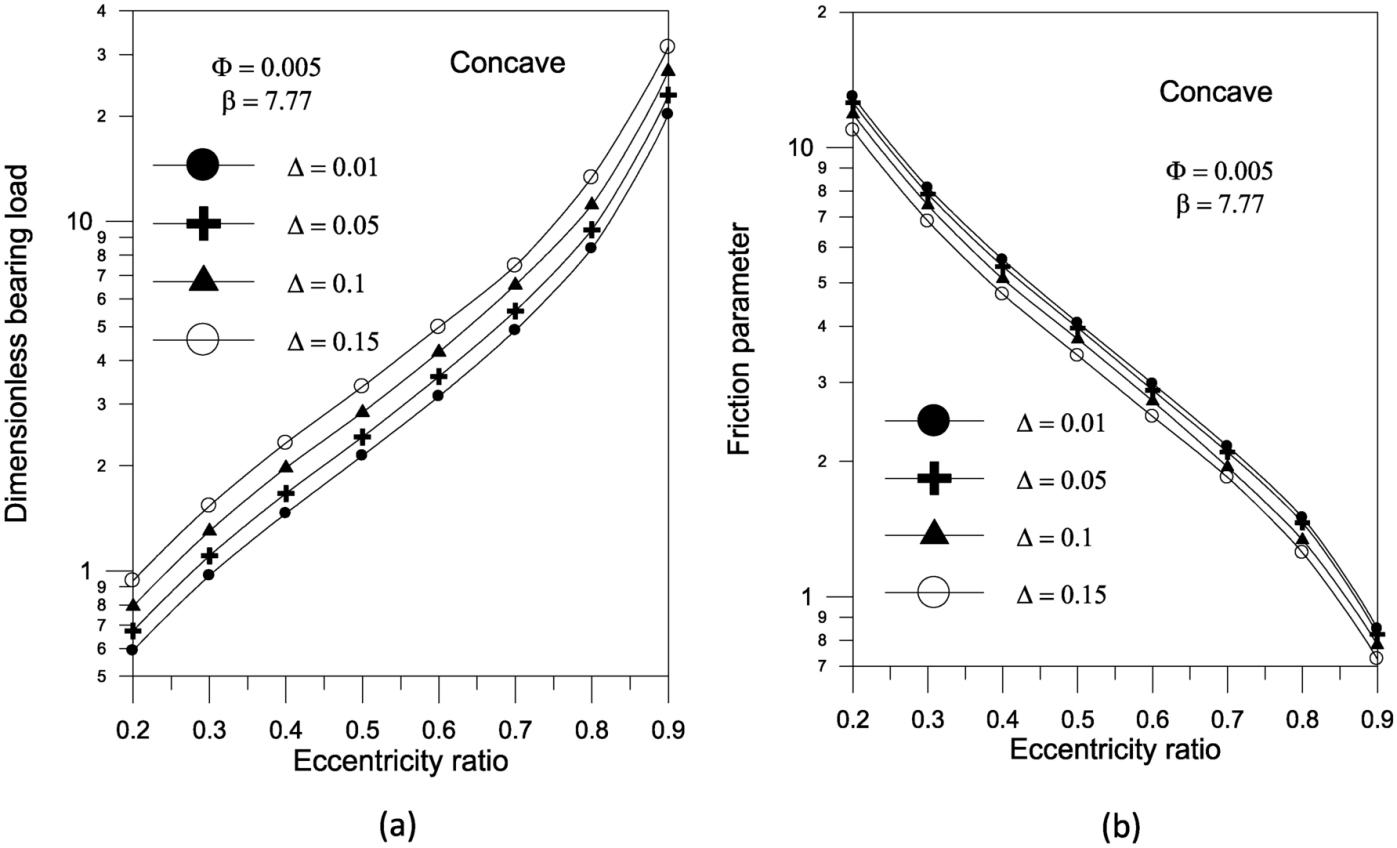
Effect of maximum axial variation on bearing characteristics (bearing load $\overline{W}$ and friction parameter $f\,(R/c)$) for concave bearing geometry at ${{\Phi = 0.005\,\,,\,\,\beta = 7.77\,\,\,and\,\,S_{o} } \mathord{\left/ {\vphantom {{\Phi = 0.005\,\,,\,\,\beta = 7.77\,\,\,and\,\,S_{o} } {D = 1}}} \right. \kern-0pt} {D = 1}}$.

**Fig. 15:**
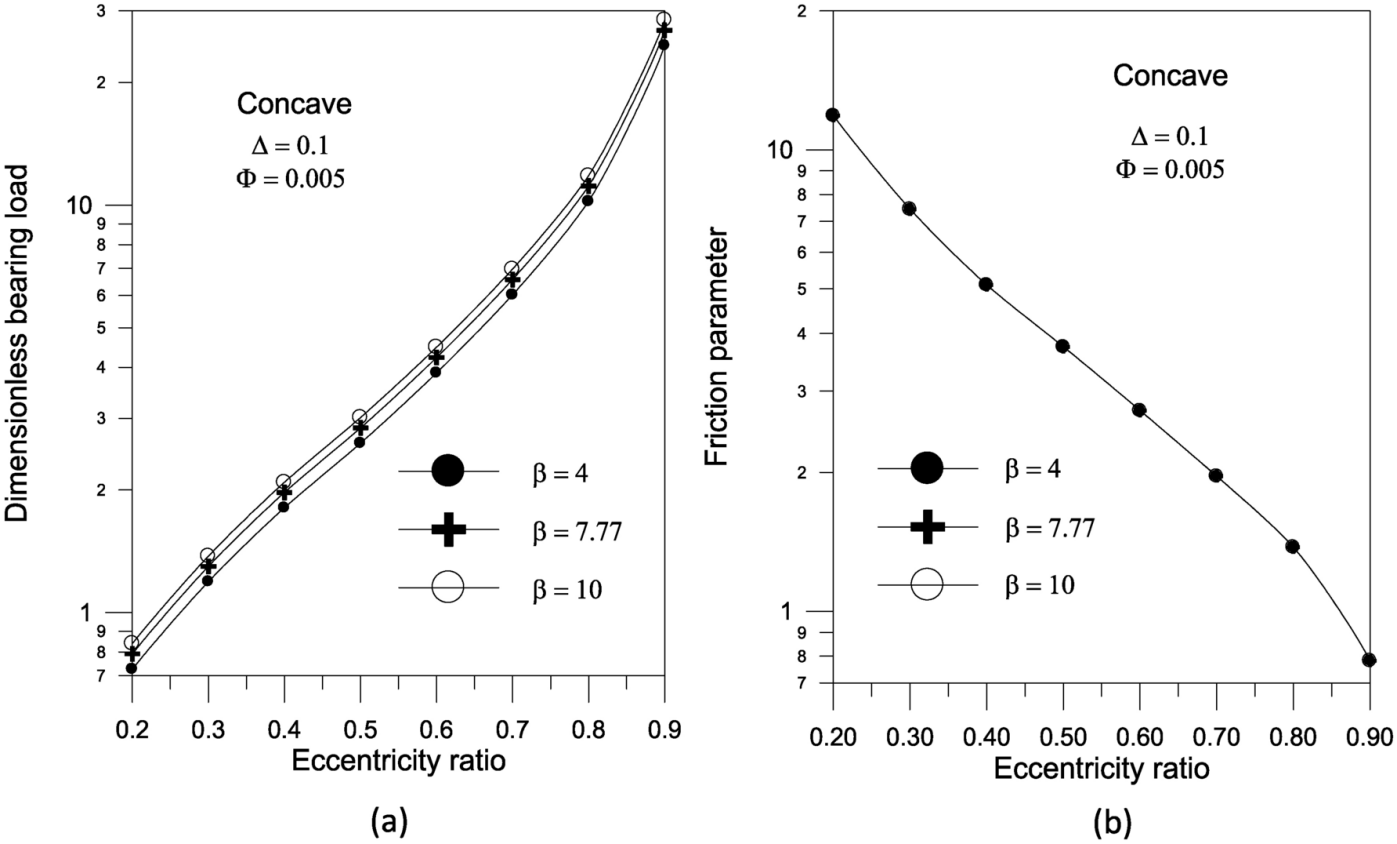
Effect of packing volume fraction on bearing characteristics (bearing load $\overline{W}$ and friction parameter $f\,(R/c)$) for concave bearing geometry at ${{\Phi = 0.005\,\,\,and\,\,S_{o} } \mathord{\left/ {\vphantom {{\Phi = 0.005\,\,\,and\,\,S_{o} } {D = 1}}} \right. \kern-0pt} {D = 1}}$.

**Fig. 16:**
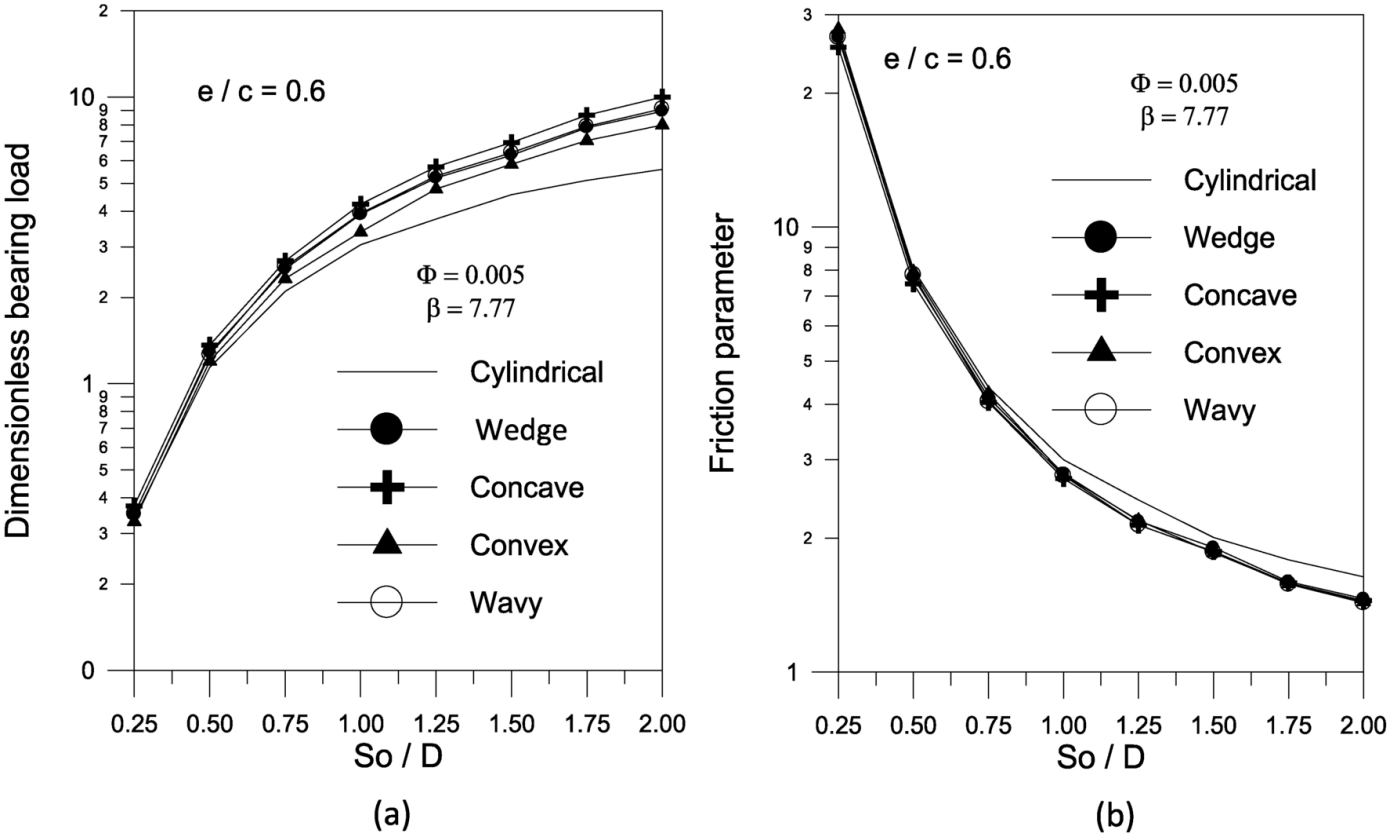
Effect of $S_{o} /D$ on bearing characteristics (bearing load $\overline{W}$ and friction parameter $f\,(R/c)$) for different bearing geometries at $\Phi = 0.005\,\,and\,\,\beta = 7.77$.

The results in Fig. 14a show that increasing the largest variation of the geometry $\Delta$ increases the bearing load capacity for concave axially curved surface. Furthermore, as shown in Fig. 14b, the largest change in the concave surface shape results in a significant reduction in the friction parameter. For a given volume fraction, the results show that increasing the aggregate packing fraction increases bearing load capacity (Fig. 15a) but has no sensible effect on friction parameter (Fig. 15b). Figure 16 shows the effect of bearing surface length to diameter ratio ${{(S_{o} } \mathord{\left/ {\vphantom {{(S_{o} } D}} \right. \kern-0pt} D})$ on bearing characteristics. The bearing load capacity increases as the length to diameter ratio increases, while the friction parameter decreases only slightly.

The relative difference in bearing load carrying capacity as a result of surface geometry, nanofluid lubricant, and the combination of the two can be introduced as follows:

$$\begin{gathered} Surface\,\,geometry\,\,\,\,\,\,:\,\,\,\,\,\,\,\,\,\,\,\,\,\,\,\,\,\,\,\,\,\,\% \,W_{g} = \frac{{W_{g} - W_{\,cyl} }}{{W_{\,cyl} }}\, \times 100 \hfill \\ Nanofluid\,\,{\text{lub}} ricant:\,\,\,\,\,\,\,\,\,\,\,\,\,\,\,\,\,\,\,\,\,\,\,\% \,W_{\,n} = \frac{{W_{gn} - W_{g} }}{{W_{g} }}\, \times 100 \hfill \\ Combination\,\,of\,\,the\,\,two:\,\,\,\,\,\,\,\,\,\,\,\,\% \,W_{\,gn} = \frac{{W_{gn} - W_{\,cyl} }}{{W_{\,cyl} }}\, \times 100 \hfill \\ \end{gathered}$$

In the same manner, the relative difference in bearing friction parameter $\% \,C_{f}$ can be estimated. Table 2 shows the relative difference in bearing characteristics caused by using a concave bearing surface shape with a dimensionless maximum axial variation $\Delta = 0.1$ and a nanofluid lubricant with a volume fraction $\Phi = 0.005$ and an aggregate packing fraction $\beta = 7.77$.

**Table 2:** Bearing characteristics for concave bearing surface shape with and without lubricant additives.

$\varepsilon$	Surface geometry		Nanofluid lubricant		Combination of the two	
	$\% \,W_{g}$	$\% \,C_{f\,g}$	$\% \,W_{n}$	$\% \,C_{f\,n}$	$\% \,W_{gn}$	$\% \,C_{f\,g\,n}$
0.2	38.412	− 9.253	16.611	− 0.003	61.404	− 9.255
0.3	38.489	− 9.303	16.611	− 0.00376	61.494	− 9.307
0.4	39.647	− 10.056	16.611	− 0.0042	62.844	− 10.06
0.5	41.553	− 11.268	16.611	− 0.0039	65.067	− 11.271
0.6	38.403	− 9.247	16.611	− 0.00294	61.394	− 9.25
0.7	43.767	− 12.635	16.611	− 0.00138	67.649	− 12.636
0.8	37.2	− 8.448	16.611	− 0.00225	59.991	− 8.45
0.9	37.191	− 8.453	16.611	− 0.0256	59.98	− 8.519
			TiO_2_ lubricant additive: ϕ = 0.005 and β = 7.77			

In comparison to plain cylindrical bearing, the results for the bearing geometries under consideration demonstrate that the presence of variations in the axial shape of the bearing increases the load carrying capacity and decreases the friction parameter (variable). Concave geometry surpasses other shapes. Furthermore, using TiO_2_ nanoparticles as a lubricant additive increases bearing load capacity while having no effect on friction parameters.

## Conclusions

An axial geometrical configuration for hydrodynamic journal bearings lubricated with noanolubricant using titanium dioxide nanoparticles as lubricant additives is proposed here. The analysis assumed that the flow in the bearing was laminar incompressible and the lubricant was isoviscous. A curvilinear coordinate system is employed to develop the Reynolds-like equation that governs the pressure inside the bearing. A theoretical investigation of the effects of increasing nanofluid aggregate sizes and nanoparticle concentrations on the static performance of journal bearings has been presented. Several conclusions are drawn:

- Compared to a simple cylindrical bearing, variations in the bearing’s axial shape increase load carrying capacity while decreasing the friction parameter. This is especially apparent with reasonably long bearings.
- Concave geometry shows superiority over other forms.
- The effect of TiO_2_ nanoparticle lubricant additives on the static characteristics of finite journal bearings is theoretically analyzed using the modified Krieger–Dougherty viscosity model. As the volume fraction and aggregate packing fraction increase, they cause a corresponding increase in the pressure distribution inside the bearing.
- The bearing load capacity increases significantly at higher values of the nanoparticle volume fraction while the friction parameter decreases. The bearing load capacity increases as the aggregate packing fraction increases with negligible effect on the friction parameter.

As a future work, it could be interesting to investigate the effect of axial geometrical variations on the stability limits of journal bearings lubricated by nanofluids.

## Data Availability

All data generated or analyzed during this study are included in this published article.

## List of symbols

$a$: Radii of primary nanoparticles, nm
$a_{a}$: Radii of aggregate nanoparticles, nm
c: Clearance in radial direction, m
$$C_{f}$$: Friction parameter, $$C_{f} = f\,\left( {{R \mathord{\left/ {\vphantom {R c}} \right. \kern-0pt} c}} \right)$$
_D_: Journal minimum diameter, m
D^*^: Fractal index
e: Eccentricity, m
f: Coefficient of friction of the bearing
F: Frictional drag force, N
$F_{n}$: Force acting normal to the journal surface, N
$\overline{F}_{f}$: Friction force in dimensionless form, $\overline{F}_{f} = {{Fc} \mathord{\left/ {\vphantom {{Fc} {\mu_{bf} \,\omega R^{2} \,S_{o} }}} \right. \kern-0pt} {\mu_{bf} \,\omega R^{2} \,S_{o} }}$
$\overline{F}_{n}$: Normal force in dimensionless form, $\overline{F}_{n} = {{F_{n} } \mathord{\left/ {\vphantom {{F_{n} } {(\mu_{bf} \,\omega R^{3} \,S_{o} /c^{2} }}} \right. \kern-0pt} {(\mu_{bf} \,\omega R^{3} \,S_{o} /c^{2} }})$
h: Fluid film thickness in direction normal to journal axis, m
$\overline{h}$: Fluid film thickness in dimensionless form, $\overline{h} = {h \mathord{\left/ {\vphantom {h c}} \right. \kern-0pt} c}$
$\overline{p}$: Dimensionless film pressure, $\overline{p} = {p \mathord{\left/ {\vphantom {p {\left( {{{\mu_{bf} \,\omega \,R^{2} } \mathord{\left/ {\vphantom {{\mu_{bf} \,\omega \,R^{2} } {c^{2} }}} \right. \kern-0pt} {c^{2} }}} \right)}}} \right. \kern-0pt} {\left( {{{\mu_{bf} \,\omega \,R^{2} } \mathord{\left/ {\vphantom {{\mu_{bf} \,\omega \,R^{2} } {c^{2} }}} \right. \kern-0pt} {c^{2} }}} \right)}}$
R: Journal minimum radius, m
$S_{o}$: Bearing curvilinear length, m
t: Time, s
$$t_{o}$$: Characteristic time $$\left( {t_{o} = {1 \mathord{\left/ {\vphantom {1 \omega }} \right. \kern-0pt} \omega }} \right)$$, s
$\overline{t}$: Time in dimensionless form, $\overline{t} = {t \mathord{\left/ {\vphantom {t {t_{o} = \omega \,t}}} \right. \kern-0pt} {t_{o} = \omega \,t}}$
$u,\,v^{\prime},\,w^{\prime}$: Velocity components in directions $x,\,y^{\prime},\,z^{\prime}$ respectively
W: Radial load normal to the journal axis, N
$W_{g}$: Load carrying capacity, with surface variation scenario, N
$W_{n}$: Load carrying capacity, with the use of a nanofluid lubricant, N
$W_{g\,n}$: Load carrying capacity, with combination of a nanofluid lubricant and surface variation, N
$\overline{W}$: Load-carrying capacity in dimensionless form, $\overline{W} = {W \mathord{\left/ {\vphantom {W {\left( {\mu_{bf} \,\omega \,R^{3} \,S_{o} /c^{2} } \right)}}} \right. \kern-0pt} {\left( {\mu_{bf} \,\omega \,R^{3} \,S_{o} /c^{2} } \right)}}$
$\overline{W}_{r} \,,\,\,\overline{W}_{t}$: Radial and transverse load components in dimensionless form with respect to $\,\left( {\mu_{bf} \,\omega \,R^{3} \,S_{o} /c^{2} } \right)$
(x, y, z): Cartesian co-ordinate system
$x,\,y^{\prime},\,z^{\prime}$: Coordinates along journal surface, normal and along journal axis respectively, m
Y, S: Coordinates normal to journal axis and along one side of the bearing surface respectively, m

## Greek symbols

β: Aggregate packing fraction, $\beta = {{a_{a} } \mathord{\left/ {\vphantom {{a_{a} } a}} \right. \kern-0pt} a}$
ε: Eccentricity ratio, $$\varepsilon = {e \mathord{\left/ {\vphantom {e c}} \right. \kern-0pt} c}$$
$\delta$: Maximum axial variation of the bearing geometry, m
$\Delta$: Dimensionless axial variation, $\Delta = {\delta \mathord{\left/ {\vphantom {\delta {S_{o} }}} \right. \kern-0pt} {S_{o} }}$
$\mu_{bf}$: Viscosity of plain engine oil, Pa s
$\mu_{nf}$: Viscosity of the nanolubricant, Pa s
$\rho$: Fluid density, kg/m^3^
$\varphi$: Attitude angle, rad
$\Phi$: Nanoparticle volume fraction
$\Phi_{m}$: Maximum packing volume fraction
$\theta$: Coordinate in tangential direction, rad
ω: Rotational angular velocity, rad/s

## References

[1] Viana, C. A. A., Alves, D. S. & Machado, T. H. Linear and nonlinear performance analysis of hydrodynamic journal bearings with different geometries. *Appl. Sci* **12**, 3215. 10.3390/app12073215 (2022).

[2] Kumar, M., Chandravansh, M. L. & Mishra, P. C. Geometrical analysis of elliptical journal bearing lubricated with Newtonian fluid. *AIP Conf. Proc.* **2341**, 020032. 10.1063/5.0050234 (2021).

[3] Bhaskera, B., Seetharamaiahb, N. & Ramesh Babu, P. Experimental investigations on elliptical journal bearing using hydrol 68 lubricating oil. In *AIP Conference Proceedings *vol. 2317, 030023. 10.1063/5.0036458 (2021).

[4] Shenglum, Z., Yu, X., Hua, X., Shiyuan, P. & Lei, Z. An experimental study on vibration suppression of adjustable elliptical journal bearing-rotor system in various vibration states. *Mech. Syst. Signal Process.* **141**, 106477. 10.1016/j.ymssp.2019.106477 (2020).

[5] Huang, B., Wang, L. & Guo, F. Performance comparison of circular, two-lobe and elliptical journal bearings based on TEHD analysis. *Ind. Lubr. Tribol.* **66**(2), 184–193. 10.1108/ILT-11-2011-0086 (2014).

[6] El-Said, A. K. H., El-Souhily, B. M., Crosby, W. A. & El-Gamal, H. A. The performance and stability of three-lobe journal bearing textured with micro protrusions. *Alex. Eng. J.* **56**, 423–432 (2017).

[7] Rao, T. V. V. L. N. et al. *Multi-lobe journal bearings analysis with limited texture* 1st edn. (CRC Press, 2021).

[8] Mehrjardi, M. Z. Dynamic stability analysis of noncircular two-lobe journal bearings with couple stress lubricant regime. *J. Eng. Tribol.* **235**(6), 1150–1167. 10.1177/1350650120945517 (2021).

[9] Jang, J. Y. & Khomsari, M. M. On the characteristics of misaligned journal bearings. *Lubricants* **3**(1), 27–53. 10.3390/lubricants3010027 (2015).

[10] Elsharkawy, A. A. Effects of misalignment on the performance of finite journal bearings lubricated with couple stress fluids. *J. Comput. Appl. Technol.* **21**(3), 137–146. 10.1504/IJCAT.2004.005939 (2004).

[11] Lombera, H. & Tello, J. I. On the finite load capacity in misaligned journal bearings. *SeMA J.* **79**, 197–320. 10.1007/s40324-021-00253-2 (2022).

[12] Girish, H. & Pai, R. Effect of journal misalignment on the static characteristics of an innovative journal bearing with adjustable elements in load-on-pad and load-between-pad configurations. *Eng. Comput.* **38**(4), 1513–1531. 10.1108/EC-03-2020-0100 (2021).

[13] Abdou, K. M. & Saber, E. Effect of rotor misalignment on stability of journal bearings with finite width. *Alex. Eng. J.* **59**, 3407–3417 (2020).

[14] Leung, P. S., Graighed, I. A. & Wilkinson, T. S. An analysis of the steady state and dynamic characteristics of a spherical hydrodynamic journal bearing. *J. Tribol.* **111**, 459–467 (1989).

[15] El-Gamal, H. A. Analysis of the steady state performance of a wedge-shaped hydrodynamic journal bearing. *Wear* **184**, 111–117 (1995).

[16] Xiaoping, P., Jin, C. & Hussain, S. H. Study on optimization of the circumferential and axial wavy geometrical congiguration of hydrodynamic journal bearing. *J. Mech. Sci. Technol.* **27**(12), 3693–3701 (2013).

[17] Tala-Ighil, N., Fillon, M. & Maspeyrot, P. Effect of textured area on the performances of a hydrodynamic journal bearing. *Tribol. Int.* **44**(3), 211–219. 10.1016/j.triboint.2010.10.003 (2011).

[18] Tala-Ighil, N., Maspeyrot, P., Fillon, M. & Bounif, A. Effects of surface texture on journal-bearing characteristics under steady-state operating conditions. *Proc. Inst. Mech. Eng. Part J: J. Eng. Tribol.* **221**(6), 623–633. 10.1243/13506501jet287 (2007).

[19] Yu, R., Chen, W. & Li, P. The analysis of elastohydrodynamic lubrication in the textured journal bearing. *Proc. Inst. Mech. Eng. Part J: J. Eng. Tribol.* **230**(10), 1197–1208. 10.1177/1350650116630207 (2016).

[20] Fesanghary, M. & Khonsari, M. M. A modification of the switch function in the elrod cavitation algorithm. *J. Tribol.* **133**(2), 024501. 10.1115/1.4003484 (2011).

[21] Sharma, S. Lubrication characteristics of newtonian-lubricated hydrodynamic bearing with partial and fully textured surface. In *Machines, Mechanism and Robotics: Proceedings of iNaCoMM 2019*. 1635–1643 (Springer, 2022). 10.1007/978-981-16-0550-5_158.

[22] Sharma, S., Jamwal, G. & Awasthi, R. K. Numerical study on steady state performance enhancement of partial textured hydrodynamic journal bearing. *Ind. Lubr. Tribol.* **71**(9), 1055–1063. 10.1108/ilt-03-2019-0083 (2019).

[23] Samuel, C., Michel, C. & Glavatskih, S. A CFD study of a finite fextured journal bearing. In *24th Symposium on Hydraulic Machinery and Systems* 1–11 (2008).

[24] Jin, H. & Zuo, W. Simulation and heat transfer calculation on a journal bearing with center circumferential groove in load zone. *Int. J. Adv. Comput. Technol.* **5**(1), 54–61 (2013).

[25] Kumar, S. C. & Ganapathi, R. CFD Analysis on hydrodynamic plain journal bearing using fluid structure interaction technique. *Int. J. Eng. Res. Technol.* **4**(7), (2015).

[26] Gertzos, K. P., Nikolakopoulos, P. G. & Papadopoulos, C. A. Lubrication by Bingham lubricant. *Tribol. Int.* **41**, 1190–1204 (2008).

[27] Shenoy, B. S., Pai, R. S., Rao, D. S. & Pai, R. Elasto-hydrodynamic lubrication analysis of full 360° journal bearing using CFD and FSI techniques. *World J. Model. Simul.* **5**(4), 315–320 (2009).

[28] Dhande, D., Pande, D. W. & Chatarkar, V. Analysis of hydrodynamic journal bearing using fluid structure interaction approach. *Int. J. Eng. Trends Technol.* **4**(8), (2013).

[29] Ouadoud, A., Mouchtachi, A. & Boutammacht, N. Hydrodynamic journal bearing. *J. Adv. Res. Mech. Eng.* **2**(1), 33–38 (2011).

[30] Mane, R. M. & Soni, S. Analysis of hydrodynamic plain journal bearing. In *The Proceedings of COMSOL Conference in Bangalore* (2013).

[31] Torgal, S. & Saini, R. Calculation of equivalent oil film temperature of journal bearing using ANSYS. *Int. J. Sci. Technol. Eng.* **1**(12), (2015).

[32] Panthi, A., Balwanshi, J., Chandravanshi, A. & Gupta, G. Design and analysis of hydrodynamic journal bearing using raimondi and boyd chart. *Int. J. Core Eng. Manag.* **2**(3), (2015).

[33] Sahu, M., Giri, A. K. & Das, A. Thermohydrodynamic analysis of a journal bearing using CFD as a tool. *Int. J. Sci. Res. Publ.* **2**(9), 2250–3153 (2012).

[34] Singla, A., Kumar, A., Bala, S., Singh, P. & Chauhan, A. Thermo-hydrodynamic analysis on temperature profile of circular bearing using computational fluid dynamics. In *Proceedings of RAECS Chandigarh* (2014).

[35] Tiwari, P. & Kumar, V. Analysis of hydrodynamic journal bearing using CFD and FSI technique. *Int. J. Eng. Res. Technol.* **3**(7), 2278–0181 (2014).

[36] Yong, H. & Balendra, R. CFD analysis on the lubrication behaviours of journal bearing with dimples. In *Proceedings of IEEE international conference on mechatronics and automation, Changchun*, China (2009).

[37] Li, Q., Zhang, S., Ma, L., Xu, W. & Zheng, S. Stiffness and damping coefficients for journal bearing using the 3D transient flow calculation. *J. Mech. Sci. Technol.* **31**(5), 2083–2091 (2017).

[38] Pengfeng, W. et al. Numerical analysis on the static performance of gas journal bearing by using finite element method. *Nanomanuf. Metrol.* **7**, 3. 10.1007/s41871-023-00219-0 (2024).

[39] Einstein, A. *Investigations on the theory of the brownian movement* (Dover Publications Inc, 1956).

[40] Brinman, H. C. The viscosity of concentrated suspensions and solution. *New J. Phys.* **20**, 571–581 (1952).

[41] Batchelor, G. K. The effect of Brownian motion on the bulk stress in a suspension of spherical particles. *J. Fluid Mech.* **83**, 97–117 (1977).

[42] Bicerano, J., Douglas, J. F. & Brune, D. A. Model for the viscosity of particle dispersions. *Polym. Rev.* **39**(4), 561–642 (1999).

[43] Krieger, I. M. A mechanism for non-Newtonian flow in suspensions of rigid spheres. *Trans. Soc. Rheol.* **3**, 137–152 (1959).

[44] Kole, M. & Dey, T. K. Effect of aggregation on the viscosity of copper oxide-gear oil nanofluids. *Int. J. Therm. Sci.* **50**(9), 1741–1747 (2011).

[45] Chen, H., Ding, Y. & Tan, C. Reological behavior of nanofluids. *New J. Phys.* **9**(10), 367–367 (2007).

[46] Mahbubul, I. M., Saidur, R. & Amalina, M. A. Latest developments on the viscosity of nanofluids. *Int. J. Heat Mass Tran.* **55**, 874–885 (2012).

[47] Ya Rudyak, V. & Krasnolutskii, S. L. Dependence of the viscosity of nanofluids on nanoparticle size and material. *Phys. Lett.* **378**, 1845–1849 (2014).

[48] Nair, K. P., Ahmed, Ms. & Al-qahtani, S. T. Static and dynamic analysis of hydrodynamic journal bearing operating under nano-lubricants. *Int. J. Nanoparticles (IJNP)* **2**, 251–262 (2009).

[49] Shenoy, B. S., Binu, K. G., Pai, R., Rao, D. S. & Pai, R. S. Effect of nanoparticle additives on the performance of an externally adjustable fluid film bearing. *Tribol. Int.* **45**(1), 38–42 (2012).

[50] Binu, K. G., Shenoy, B. S., Rao, D. S. & Pai, R. A variable viscosity approach for the evaluation of load carrying capacity of oil lubricated journal bearing with TiO_2_ nanoparticles as lubricant additives. *Procedia Mater. Sci.* **6**, 1051–1067 (2014).

[51] Babu, K. S., Nair, K. P. & Rajendrakumar, P. K. Computational analysis of journal bearing operating under lubricant containing Al_2_O_3_ and ZnO nanoparticles. *Int. J. Eng. Sci. Technol.* **6**(1), 34–42 (2014).

[52] Rao, T. V. V. N., Rani, A. M. A., Sufian, S. & Mohamed, N. M. Thin film hydrodynamic bearing analysis using nanoparticle additive lubricant. In *Engineering applications of nanotechnology* 149–173 (Springer, 2017). 10.1007/978-3-319-29761-3.

[53] Wang, X. L., Zhu, K. Q. & Wen, S. Z. Thermohydrodynamic analysis of journal bearings lubricated with couple stress fluids. *Tribol. Int.* **34**, 335–343 (2001).

[54] Wang, X. L., Zhu, K. Q. & Gui, C. L. A study of a journal bearing lubricated by couple stress fluids considering thermal and cavitation effects. *J. Eng. Tribol.* **216**(Part J), 293–305 (2002).

[55] Guha, S. K. A theoretical analysis of dynamic characteristics of finite hydrodynamic journal bearings lubricated with couple stress fluids. *Proc. Inst. Mech. Eng. Part J J. Eng. Tribol.* **218**, 125–133 (2004).

[56] Crosby, W. A. & Chetti, B. The static and dynamic characteristics of a two-lobe journal bearing lubricated with couple-stress fluid. *Tribol. Trans.* **52**, 262–268 (2009).

[57] Mokhiamer, U. A., Crosby, W. A. & El-Gamal, H. A. Study of a journal bearing lubricated by fluids with couple stress considering the elasticity of the liner. *Wear* **224**, 194–201 (1999).

[58] Ibhadode, A. O. Elastohydrodynamic analysis of an offsed journal bearing lubricated with couple stress fluid. *Int. J. Eng. Res. Afr.* **2**, 53–62 (2010).

[59] Dass, T., Gunakala, S. R. & Comissiong, D. M. G. The combined effect of couple stress, variable viscosity and velocity-slip on the lubrication of finite journal bearings. *Ain Shams Eng. J.* **11**, 501–518 (2020).

[60] Zhu, J., Qian, H., Wen, H., Zeng, L. & Zhu, H. Analysis of misaligned journal bearing lubrication performance considering the effect of lubricant couple stress and shear thinning. *J. Mech.* **37**, 282–290 (2021).

[61] Awad, H., Abdou, K. M. & Saber, E. Steady state characteristics and stability limits of oil lubricated journal bearings using titanium dioxide nanoparticles as lubricant additives. *Results Eng.* **20**, 101486 (2023). 10.1038/s41598-025-97948-7 PMC12053646 40325062

[62] Microsoft Corporation. Fortran PowerStation 4.0. Microsoft, 1996. Available at: https://archive.org/details/ms-fortran-40-powerstation-standard

